# Supplementary material for: Integrated pharmaco-proteogenomics defines two subgroups in isocitrate dehydrogenase wild-type glioblastoma with prognostic and therapeutic opportunities
Source: Nat Commun. 2020 Jul 3;11:3288. doi: 10.1038/s41467-020-17139-y (PMC7335111; doi:10.1038/s41467-020-17139-y)
Supplement: Supplementary file 1 — Supplementary Information [file 41467_2020_17139_MOESM1_ESM.pdf]

“Integrated pharmaco-proteogenomics defines two subgroups in isocitrate dehydrogenase wild-type glioblastoma with prognostic and therapeutic opportunities.”

Oh et al., 2020

Supplementary Information

Supplementary Figures 1 ~ 6 (page 1 to 5)

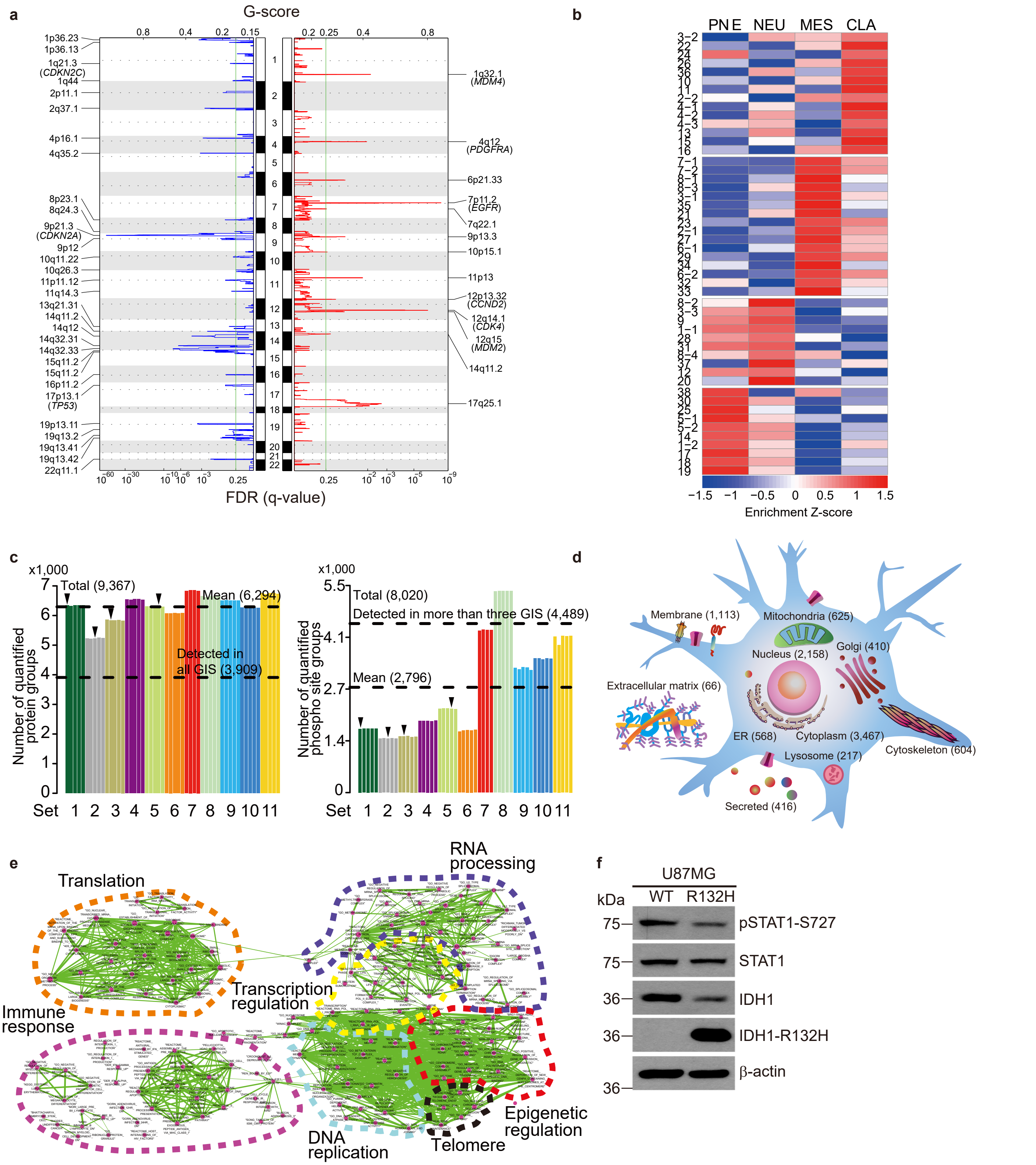

Supplementary Figure 1. Proteogenomic characterization of glioblastoma multiforme (GBM).
a. Copy number alterations in GBM samples - amplification (red) and deletion (blue). The top x-axis indicates the G-scores from the GISTIC analysis. The bottom x-axis indicates the false discovery rate (FDR). The y-axis represents the chromosomal locations. The green lines represent an FDR of 0.25, and the candidate genes therein are shown in parentheses. **b**. RNA subtyping of GBM samples. Heatmap representation of the single-sample gene molecular set enrichment score (ssGSEA) of each of the four RNA subtypes per glioma sample. **c**. Number of quantified protein groups and phosphorylated site groups in 54 samples (multiplexed in 11 sets). (Left) The upper and lower dashed lines indicate the average number of detected proteins (N = 6,294) and the number of detected proteins in all GISs (N = 3,909), respectively. Normal samples are indicated above as inverted triangles. See Supplementary Data 2 for the complete quantification data. (Right) The dashed line indicates the average number of quantified phosphorylated site groups (N = 2,796). **d**. Subcellular distribution of global proteins detected in all GIS. A total of 3,909 unique global proteins (corresponding to 4,079 canonical UniProt IDs) were annotated by the human compartment DB (score ≥ 3) and UniProt Knowledgebase by allowing redundancy. **e.** Gene set analysis of proteins elevated in glioma tissues. The over-represented gene sets (FDR < 1%) were visualized using EnrichmentMap with default parameters. The connected gene sets were manually annotated as indicated, based on the corresponding function. See Supplementary Data 3 for further details. **f**. Comparison of STAT1 (pS727) levels in U87MG isogenic cell lines (N = 2). Molecular weight markers (kDa) are shown on the left. WT: wild-type. Source data are provided as a Source Data file.

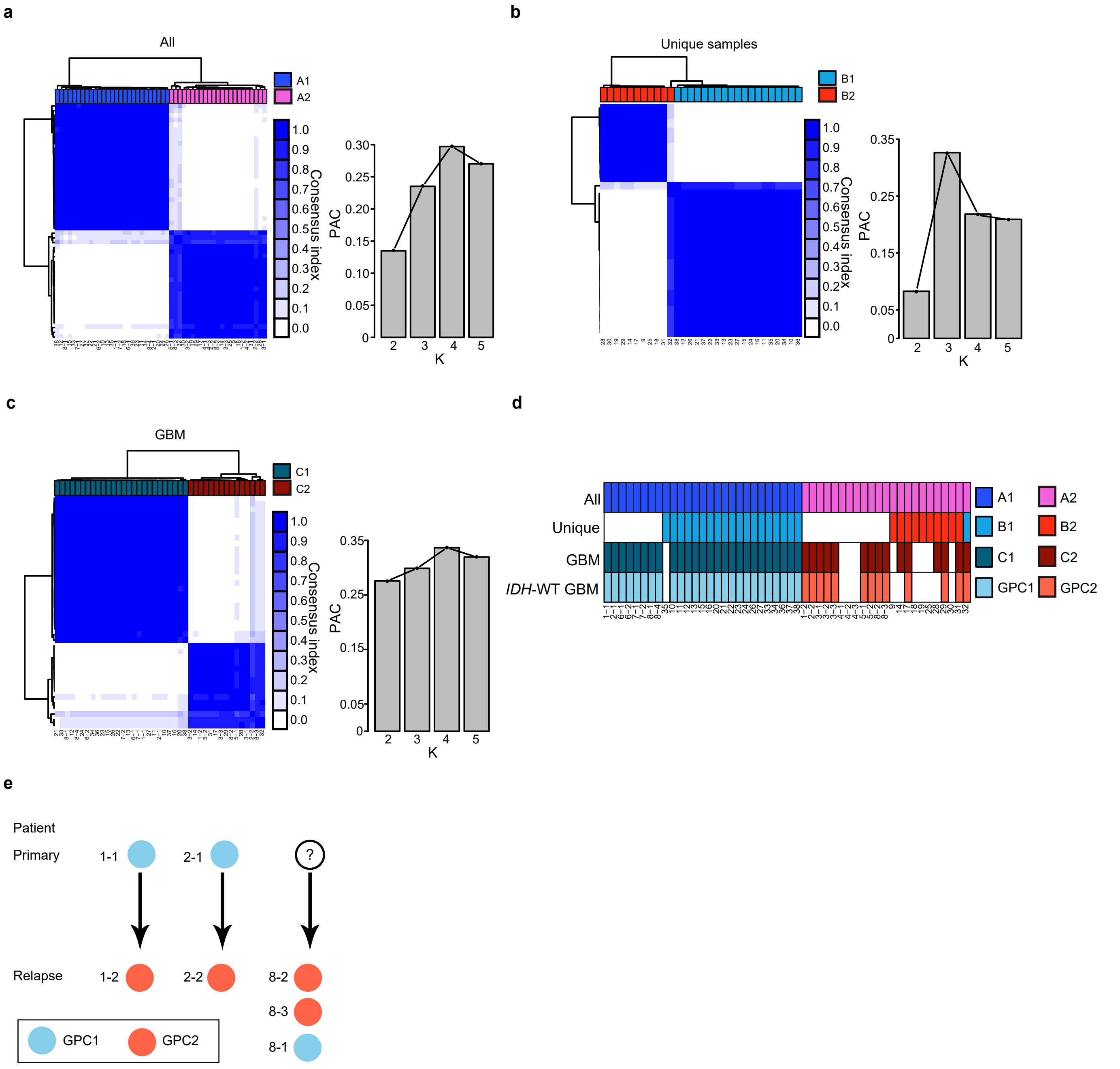

Supplementary Figure 2. Robustness of proteomic subtypes of *IDH* wild-type GBM and subtype-switch in relapse samples.
a. Consensus clustering with 50 glioma samples. (Left) The heatmap shows the consensus score. (Right) The bar chart represents the proportion of ambiguous clustering (PAC) for the indicated K values. The number of clusters (K) with the lowest PAC score is considered the optimal cluster number. **b.** Consensus clustering with 30 unique samples. **c.** Consensus clustering with 41 GBM samples. **d.** Comparison of consensus clustering results from all glioma samples, unique glioma samples, GBM samples and *IDH* wild-type GBM samples. **e.** Subtype-switch observed in paired primary and relapse samples. Each column represents a patient, and each circle within a column represents separate primary and relapse tumor samples obtained for that patient. Primary GBM tissue was missing for patient 8. Source data are provided as a Source Data file.

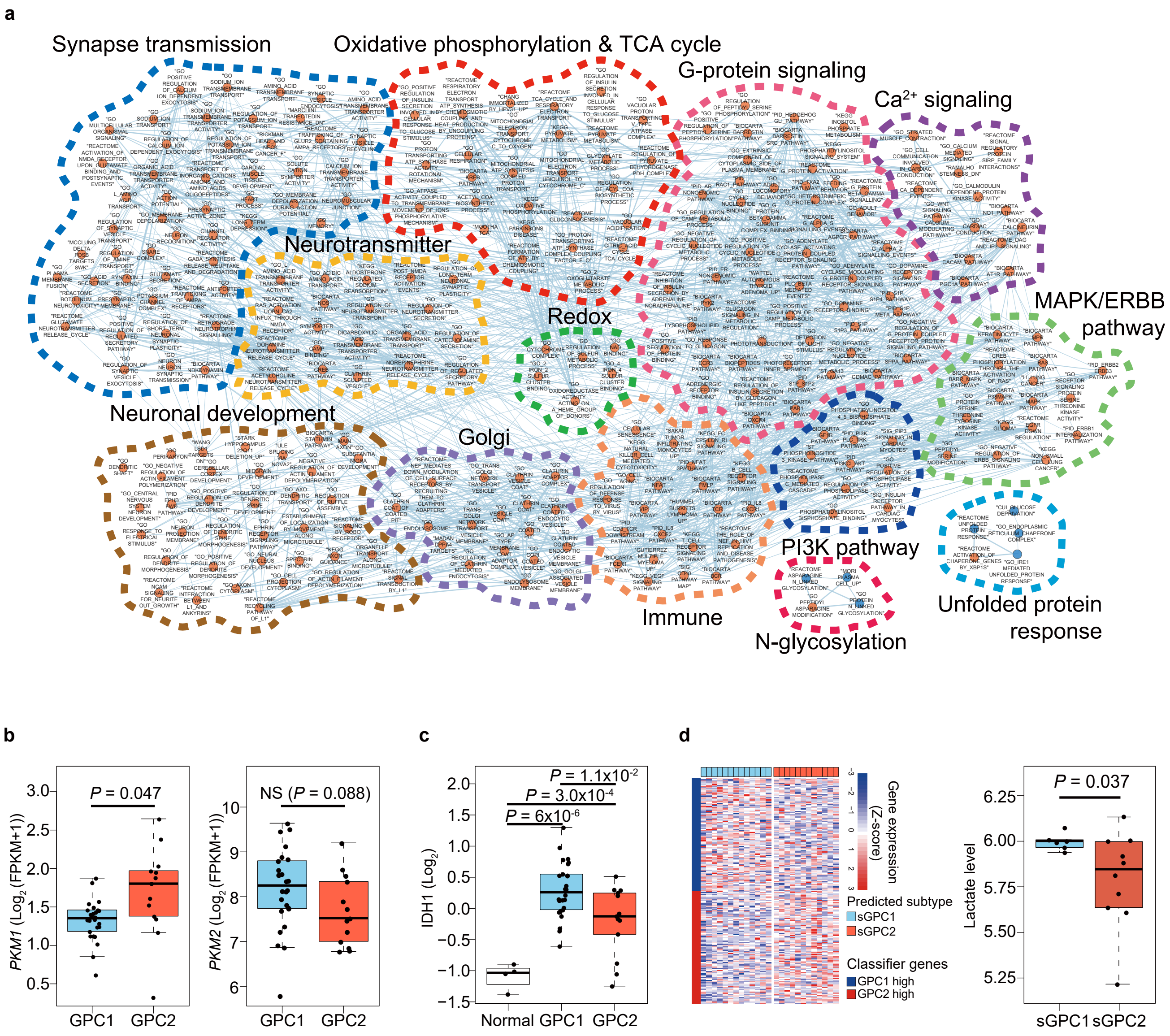

Supplementary Figure 3. Characterization of IDH wild-type GBM proteomic subtypes.

a. Gene set enrichment analysis of the top 10% loading proteins of the first principal component determining GBM proteome subtypes. The over-represented gene sets (FDR < 10%; hypergeometric test) were visualized by EnrichmentMap with default parameters. **b.** Subtype-specific mRNA expression of PKM isoforms. The box-and-whisker plots represent the medians (middle line), first quartiles (lower bound line), third quartiles (upper bound line) and the ±1.5 x interquartile ranges (whisker lines); the raw data are overlaid. *P* values (two-sided unpaired Student's t-test) are displayed for comparisons between GPC1 (N = 26) and GPC2 tumors (N = 13). **c.** Comparison of IDH1 expression between tumor subtypes and nearby normal samples. The box-and-whisker plots, tests, and sample size are the same as in b. **d.** Classification of 47 GBM cell lines into gene expression-based surrogate GPC subtypes. The heatmap displays gene expression levels of classifier genes (left). Comparison of lactate levels in GBM cell lines according to sGPC subtype (right). The description of the box-and-whisker plots is the same as in b. The numbers of samples in sGPC1 and sGPC2 are 6 and 10, respectively. *P* values were obtained by two-sided unpaired Student's t-test. Source data are provided as a Source Data file.

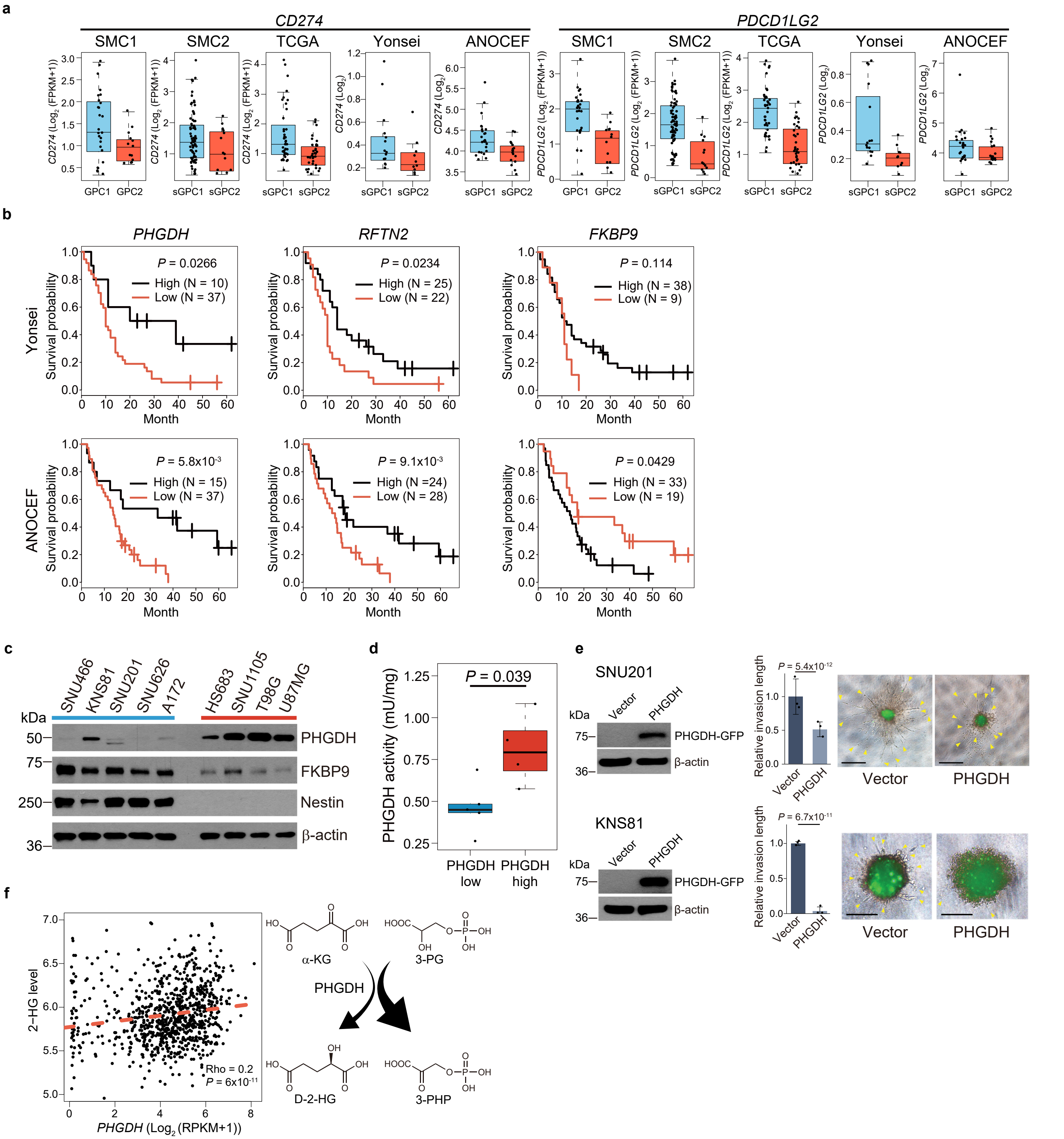

Supplementary Figure 4. PHGDH predicts a favorable prognosis in *IDH* wild-type GBM.

a. Subtype-specific expression of *CD274* (PD-L1) and *PDCD1LG2* (PD-L2) in the five datasets. The box-and-whisker plots represent the medians (middle line), first quartiles (lower bound line), third quartiles (upper bound line), and the ±1.5 x interquartile ranges (whisker lines). The raw data are overlaid. **b.** Kaplan-Meier plots for three indicated prognostic markers in the Yonsei (upper panels) and ANOCEF (lower panels) cohorts. Patients were classified by the same method used in Fig. 4e. *P* values were obtained by the two-sided log-rank test. **c.** Steady state accumulation of the indicated proteins was assessed by immunoblotting of whole cell lysates from the indicated GBM cell lines. Molecular weight markers (kDa) are shown on the left. (N = 2) **d.** Comparison of PHGDH enzymatic activity. See Methods for details. The box-and-whisker plots represent the medians (middle line), first quartiles (lower bound line), third quartiles (upper bound line), and the ±1.5 x interquartile ranges (whisker lines). The raw data are overlaid. *P* values were calculated by two-sided unpaired Student's t-test. The numbers of samples in PHGDH-low and high groups are 5 and 4, respectively. **e.** PHGDH levels by immunoblotting (left panels, N = 2), relative invasion lengths of the tumorspheres (middle panels), and representative images of 3D invasions (right panels) in SNU201 (upper panels) and KNS81 (lower panels)-derived tumor spheres after 96 hours of transfection of plasmids (empty vector or PHGDH cDNA). Arrowheads indicate invasive fronts. Molecular weight markers (kDa) are shown on the left. Two-way ANOVA was used for the comparison of relative invasion length between transfection groups. Error bars indicate ±SD, n = 3. Scale bar : 500μm (SNU201), 200μm (KNS81). **f.** Correlation between *PHGDH* expression and 2-hydroxyglutarate (2-HG) levels in 878 IDH wild-type cancer cell lines. Gene expression and 2-HG abundance data were obtained from the CCLE database. Rho and *P* values were obtained by Spearman's correlation test. (Right) Schematic drawing of the primary (thick arrow) and promiscuous (thin arrow) function of PHGDH. Source data are provided as a Source Data file.

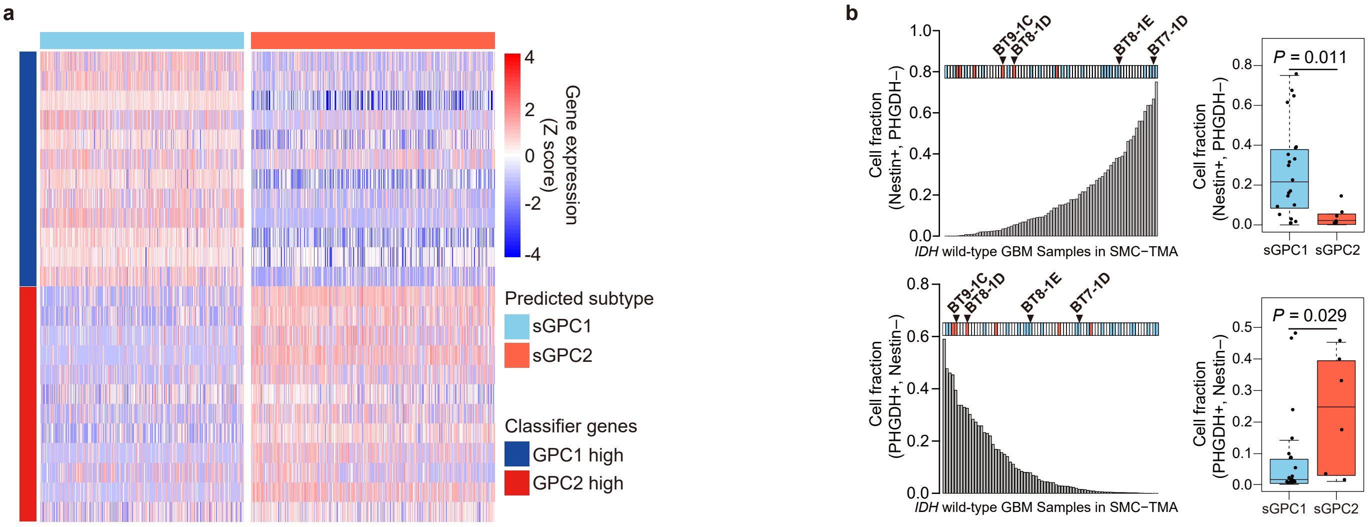

Supplementary Figure 5. Characterization of GBM single-cells.
a. Classification of single cells into surrogate GPC subtypes. The heatmap displays the expression levels of surrogate classifier genes and the predicted subtypes (upper color stripe) for single cells in the dataset generated by Darmanis et al., 2017. The single cells were sorted according to their permutation *P* value. **b.** Fraction of Nestin+/PHGDH- (Upper left panel) and PHGDH+/Nestin- (Lower left panel) cells in GBM tissues from SMC-TMA cohort. sGPC-subtype for each sample is indicated above (left panels). Samples used in Fig. 5g were denoted by black arrowheads. Comparison of the cell fractions between sGPC1- (N = 21) and sGPC2-subtype samples (N = 6) (right panels). The box-and-whisker plots represent the medians (middle line), first quartiles (lower bound line), third quartiles (upper bound line), and the ±1.5 x interquartile ranges (whisker lines). The raw data are overlaid. Statistical significance of sGPC1-subtype dependent elevations of Nestin+/PHGDH- cell fractions and sGPC2-subtype dependent elevation of PHGDH+/Nestin- cell fractions was evaluated by one-sided unpaired Wilcoxon rank-sum test. Source data are provided as a Source Data file.

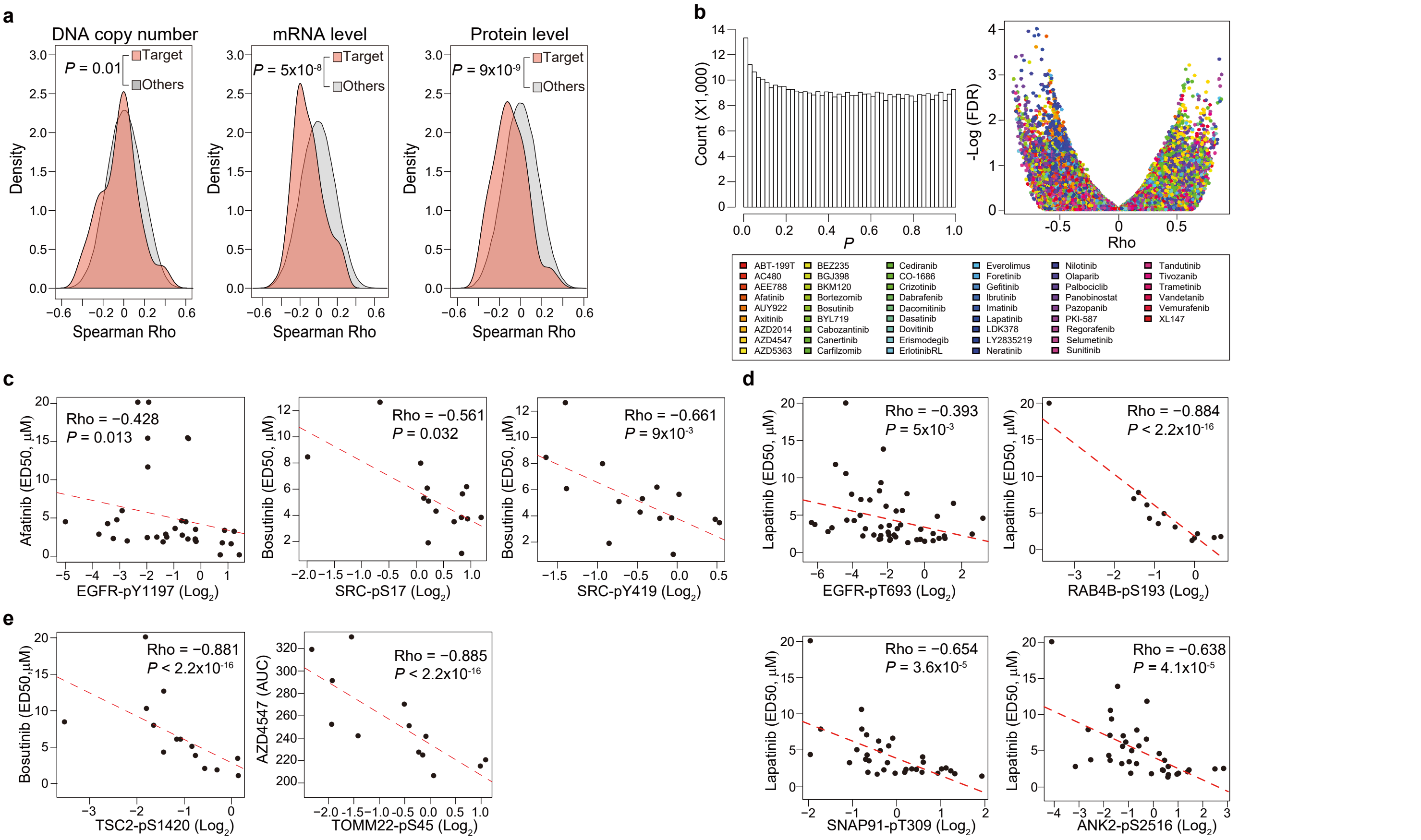

Supplementary Figure 6. An integrative analysis of pharmacogenomic data from matched primary GBM cells.
a. Density plots for drug response-target correlations at the DNA copy number, RNA and protein levels. Genes with available protein data were used for comparison. *P* values were obtained by two-sided unpaired Wilcoxon rank-sum test. **b.** Correlation tests between the drug response (AUC and ED50) and the abundance of phosphoproteins. (Left) Histogram of *P*-values. (Right) FDR score distribution. *P* values were obtained by Spearman's correlation test.**c.** Correlation between phosphoprotein levels and the response to the EGFR inhibitor afatinib and SRC inhibitor bosutinib. *P* values were obtained by Spearman's correlation test. **d.** Correlation between phosphoprotein levels and the response to the EGFR-HER2 inhibitor lapatinib. *P* values were obtained by Spearman's correlation test. **e.** Correlation between phosphoprotein levels and the response to bosutinib and FGFR inhibitor AZD4547. *P* values were obtained by Spearman's correlation test. Source data are provided as a Source Data file.
