# Supplementary material for: Integrated pharmaco-proteogenomics defines two subgroups in isocitrate dehydrogenase wild-type glioblastoma with prognostic and therapeutic opportunities
Source: Nat Commun. 2020 Jul 3;11:3288. doi: 10.1038/s41467-020-17139-y (PMC7335111; doi:10.1038/s41467-020-17139-y)
Supplement: Supplementary file 3 — Description of Additional Supplementary Information [file 41467_2020_17139_MOESM3_ESM.pdf]

Description of Additional Supplementary Files

File Name: Supplementary Data 1
Description: Clinical and molecular annotation of the SMC cohorts 1 and 2 and TMA.

File Name: Supplementary Data 2
Description: Protein abundance profile of each sample. Values represent log2 (sample intensity/GIS intensity).

File Name: Supplementary Data 3
Description: List of differentially expressed proteins and pathways. The *P* values of differentially expressed proteins and phosphoproteins were obtained by two-sided unpaired Student's t-test.

File Name: Supplementary Data 4
Description: Univariate Cox regression analysis of 271 proteins in *IDH* wild-type GBM.

File Name: Supplementary Data 5
Description: Short tandem repeat profiles of GBM cell lines.

File Name: Supplementary Data 6
Description: Area under the viability curve (AUC) values and median effective doses (ED50 in μM) for each of the GBM patient-derived cells treated with 51 drugs.
