# Supplementary material for: Integrated pharmaco-proteogenomics defines two subgroups in isocitrate dehydrogenase wild-type glioblastoma with prognostic and therapeutic opportunities
Source: Nat Commun. 2020 Jul 3;11:3288. doi: 10.1038/s41467-020-17139-y (PMC7335111; doi:10.1038/s41467-020-17139-y)
Supplement: Supplementary file 10 — Reporting summary [file 41467_2020_17139_MOESM10_ESM.pdf]

nature research

Corresponding author(s): Hyun Seok Kim

Last updated by author(s): June 2nd, 2020

Reporting Summary

Nature Research wishes to improve the reproducibility of the work that we publish. This form provides structure for consistency and transparency in reporting. For further information on Nature Research policies, see Authors & Referees and the Editorial Policy Checklist .

Statistics

For all statistical analyses, confirm that the following items are present in the figure legend, table legend, main text, or Methods section.

n/a	Confirmed	
☐	☒	The exact sample size (*n*) for each experimental group/condition, given as a discrete number and unit of measurement
☐	☒	A statement on whether measurements were taken from distinct samples or whether the same sample was measured repeatedly
☐	☒	The statistical test(s) used AND whether they are one- or two-sided<br>*Only common tests should be described solely by name; describe more complex techniques in the Methods section.*
☐	☒	A description of all covariates tested
☐	☒	A description of any assumptions or corrections, such as tests of normality and adjustment for multiple comparisons
☐	☒	A full description of the statistical parameters including central tendency (e.g. means) or other basic estimates (e.g. regression coefficient) AND variation (e.g. standard deviation) or associated estimates of uncertainty (e.g. confidence intervals)
☐	☒	For null hypothesis testing, the test statistic (e.g. *F*, *t*, *r*) with confidence intervals, effect sizes, degrees of freedom and *P* value noted<br>*Give P values as exact values whenever suitable.*
☒	☐	For Bayesian analysis, information on the choice of priors and Markov chain Monte Carlo settings
☒	☐	For hierarchical and complex designs, identification of the appropriate level for tests and full reporting of outcomes
☐	☒	Estimates of effect sizes (e.g. Cohen's *d*, Pearson's *r*), indicating how they were calculated

Our web collection on statistics for biologists contains articles on many of the points above.

Software and code

Policy information about availability of computer code

Data collection	mRNA expression and clinical data of TCGA-GBM were downloaded using TCGAbiolinks R packages (version 2.6.12). In case of single-cell data, we downloaded single-cell mRNA expression and meta data from http://gbmseq.org.
Data analysis	R (version 3.5.3)<br>R script (https://github.com/hk-lab-software/gbm2020/)<br>BWA (version 0.6.2)<br>Picard (version 1.73)<br>SAMtools (version 0.1.18)<br>GATK (version 2.5-2)<br>GSNAP (version 2012-12-20)<br>STAR (version 2.5.4b)<br>Cufflinks (version 2.0.2)<br>Genome analysis toolkit (version 2.5-2)<br>MuTect (version 1.1.4)<br>SomaticIndelDetector (GATK version 2.2)<br>Variant Effect Predictor (version 37.75)<br>MaxQuant (version 1.5.6.0)<br>edgeR package (version 3.20.9)<br>DNAcopy package (version 1.52.0)<br>TCGAbiolinks (version 2.6.12)<br>CellSence (version 1.15)<br>ImageJ (version 1.52a)<br>drc package (version 3.0-1)

inForm AIA (version 2.2)
MS-GF+ (version 2014.03.26)
Cytoscape (version 3.7.1)
Bedtools (version 2.26.0)

For manuscripts utilizing custom algorithms or software that are central to the research but not yet described in published literature, software must be made available to editors/reviewers. We strongly encourage code deposition in a community repository (e.g. GitHub). See the Nature Research guidelines for submitting code & software for further information.

Data

Policy information about availability of data

All manuscripts must include a data availability statement. This statement should provide the following information, where applicable:

- Accession codes, unique identifiers, or web links for publicly available datasets
- A list of figures that have associated raw data
- A description of any restrictions on data availability

The data supporting the findings of this manuscript are available from the corresponding author upon reasonable request. Previously published RNA-Seq data that were reanalyzed here are available under accession code EGAS00001002515. The mass spectrometry proteomics data have been deposited to the ProteomeXchange Consortium via the PRIDE partner repository with the dataset identifier PXD015545. The mass spectrometry raw files were searched against the Swiss-Prot human database (released in March 2014; http://www.uniprot.org) using the Andromeda search engine included in MaxQuant. Microarray data of Yonsei cohort (GSE131837) and ANOCEF (E-TABM-898) was downloaded from GEO and ArrayExpress, respectively. Gene expression data of TCGA were downloaded from https://portal.gdc.cancer.gov. The source data underlying Figs. 4g and Supplementary Figs. 1f and 4c/d/e are provided as a compressed source data file.

Field-specific reporting

Please select the one below that is the best fit for your research. If you are not sure, read the appropriate sections before making your selection.

☒ Life sciences ☐ Behavioural & social sciences ☐ Ecological, evolutionary & environmental sciences

For a reference copy of the document with all sections, see nature.com/documents/nr-reporting-summary-flat.pdf

Life sciences study design

All studies must disclose on these points even when the disclosure is negative.

Sample size	Sample size was determined solely by the availability of glioma samples in our cohort.
Data exclusions	No data was excluded.
Replication	All the experiments were replicated and indicated in the Methods and Figure Legends. All attempts at replication were successful.
Randomization	Randomization is not relevant to our study. GPC1 and GPC2 groups were determined based on sample specific proteome data.
Blinding	Blinding is not relevant to our study. GPC1 and GPC2 groups were determined based on sample specific proteome data.

Reporting for specific materials, systems and methods

We require information from authors about some types of materials, experimental systems and methods used in many studies. Here, indicate whether each material, system or method listed is relevant to your study. If you are not sure if a list item applies to your research, read the appropriate section before selecting a response.

Materials & experimental systems

n/a	Involved in the study	
☐	☒	Antibodies
☐	☒	Eukaryotic cell lines
☒	☐	Palaeontology
☒	☐	Animals and other organisms
☐	☒	Human research participants
☒	☐	Clinical data

Methods

n/a	Involved in the study	
☒	☐	ChIP-seq
☒	☐	Flow cytometry
☒	☐	MRI-based neuroimaging

Antibodies

Antibodies used	Immuno blot:<br>STAT1 (Cell signaling technology, #80916, D1K9Y, 1/1000)<br>pSTAT1 Serine-727 (Cell signaling technology, #8826, D3B7, 1/1000)

	PHGDH (Cell signaling technology, #66350, D8F3O, 1/1000)<br>NES (Abcam, ab105389, 1/1000)<br>FKBP9 (Novus Biologicals, NBP1-83887, 1/1000)<br>β-actin (Cell signaling technology, # 4970, 13E5, 1/1000)<br>IDH1-R132H (DIANOVA, DIA-H09, 1/1000)<br>IDH1 (Cell signaling technology, #8137, D2H1, 1/1000)<br>Anti-rabbit IgG (Jackson immunoresearch, 111-035-144, 1/5000)<br>Anti-mouse IgG (Jackson immunoresearch, 115-035-146, 1/5000)<br><br>Immunohistochemistry (Opal)<br>PHGDH (Atlas Antibodies, HPA021241, 1/1000)<br>NES (Atlas Antibodies, HPA007007, 1/700)<br>HRP Ms+Rb secondary Ab 1x solution (Akoya Biosciences, ARH1001EA)
Validation	Primary antibodies used in this study have been validated by the respective companies and numerous research groups.<br>Validation links are listed below;<br>STAT1 : https://www.cellsignal.com/products/primary-antibodies/stat1-d1k9y-rabbit-mab/14994<br>pSTAT1 Serine-727 : https://www.cellsignal.com/products/primary-antibodies/phospho-stat1-ser727-d3b7-rabbit-mab/8826<br>PHGDH : https://www.cellsignal.com/products/primary-antibodies/phgdh-d8f3o-rabbit-mab/66350<br>NES : https://www.abcam.com/nestin-antibody-sp103-ab105389.html<br>FKBP9 : https://www.novusbio.com/products/fkbp9-antibody_nbp1-83887<br>β-actin : https://www.cellsignal.com/products/primary-antibodies/b-actin-13e5-rabbit-mab/4970<br>IDH1-R132H : https://www.dianova.com/en/shop/dia-h09-anti-idh1-r132h-hu-from-mouse-h09-unconj/<br>IDH1 : https://www.cellsignal.com/products/primary-antibodies/idh1-d2h1-rabbit-mab/8137<br>Anti-rabbit IgG : https://www.citeab.com/antibodies/2036616-111-035-144-peroxidase-affinipure-goat-anti-rabbit-i<br>Anti-mouse IgG : https://www.citeab.com/antibodies/2036653-115-035-146-peroxidase-affinipure-goat-anti-mouse-ig<br><br>Immunohistochemistry (Opal)<br>PHGDH : https://www.atlasantibodies.com/products/antibodies/primary-antibodies/triple-a-polyclonals/phgdh-antibody-hpa021241/<br>NES : https://www.atlasantibodies.com/products/antibodies/primary-antibodies/triple-a-polyclonals/nes-antibody-hpa007007/<br>HRP Ms+Rb secondary Ab 1x solution : https://www.nature.com/articles/s41598-019-42986-1

Eukaryotic cell lines

Policy information about cell lines

Cell line source(s)	KNS81 cells were purchased from the JCRB cell bank. U87MG and U87MG-IDH1-R132H cells were purchased from the ATCC. SNU466, SNU201, SNU626, A172, HS683, SNU1105, and T98G were purchased from Korean Cell Line Bank (KCLB).
Authentication	All the cell lines were authenticated by STR fingerprints.
Mycoplasma contamination	The absence of mycoplasma contamination was confirmed in all cell lines by using e-Myco VALiD Mycoplasma PCR detection kit (LiliF, cat. no. 25299).
Commonly misidentified lines<br>(See ICLAC register)	Although U87MG is reported as a misidentified cell line in the ICLAC database, cell identity does not affect the conclusion of the experiment since the experimental goal was to investigate the IDH1-mutation's effect on STAT1 phosphorylation in an isogenic cell context. Please see Supplementary Fig 1f, where we confirmed the IDH1 mutation status of U87MG (ICLAC-00535, ATCC) and its IDH1-R132H knock-in derivative by immunoblot analysis.

Human research participants

Policy information about studies involving human research participants

Population characteristics	Tumor specimens and the corresponding clinical records were obtained from patients who underwent surgical resection at the Samsung Medical Center (SMC) and who had provided informed consent. Participants had an average age of 54 ± 26 years with 54% female and 16.2% of IDH1 mutation. All participants were received radiotherapy, as reported in Supplementary Data1.
Recruitment	Glioblastoma patients participated in this study voluntarily after appropriate consent was obtained. There was no selection bias with regard to age, gender, mutation status etc. while recruiting the patients for this study.
Ethics oversight	This study was approved by the Samsung Medical Center (SMC) Institutional Review Board (IRB #201004004 and 200504001).

Note that full information on the approval of the study protocol must also be provided in the manuscript.
