# Supplementary figures and images for: Integrated pharmaco-proteogenomics defines two subgroups in isocitrate dehydrogenase wild-type glioblastoma with prognostic and therapeutic opportunities
Source: Nat Commun. 2020 Jul 3;11:3288. doi: 10.1038/s41467-020-17139-y (PMC7335111; doi:10.1038/s41467-020-17139-y)

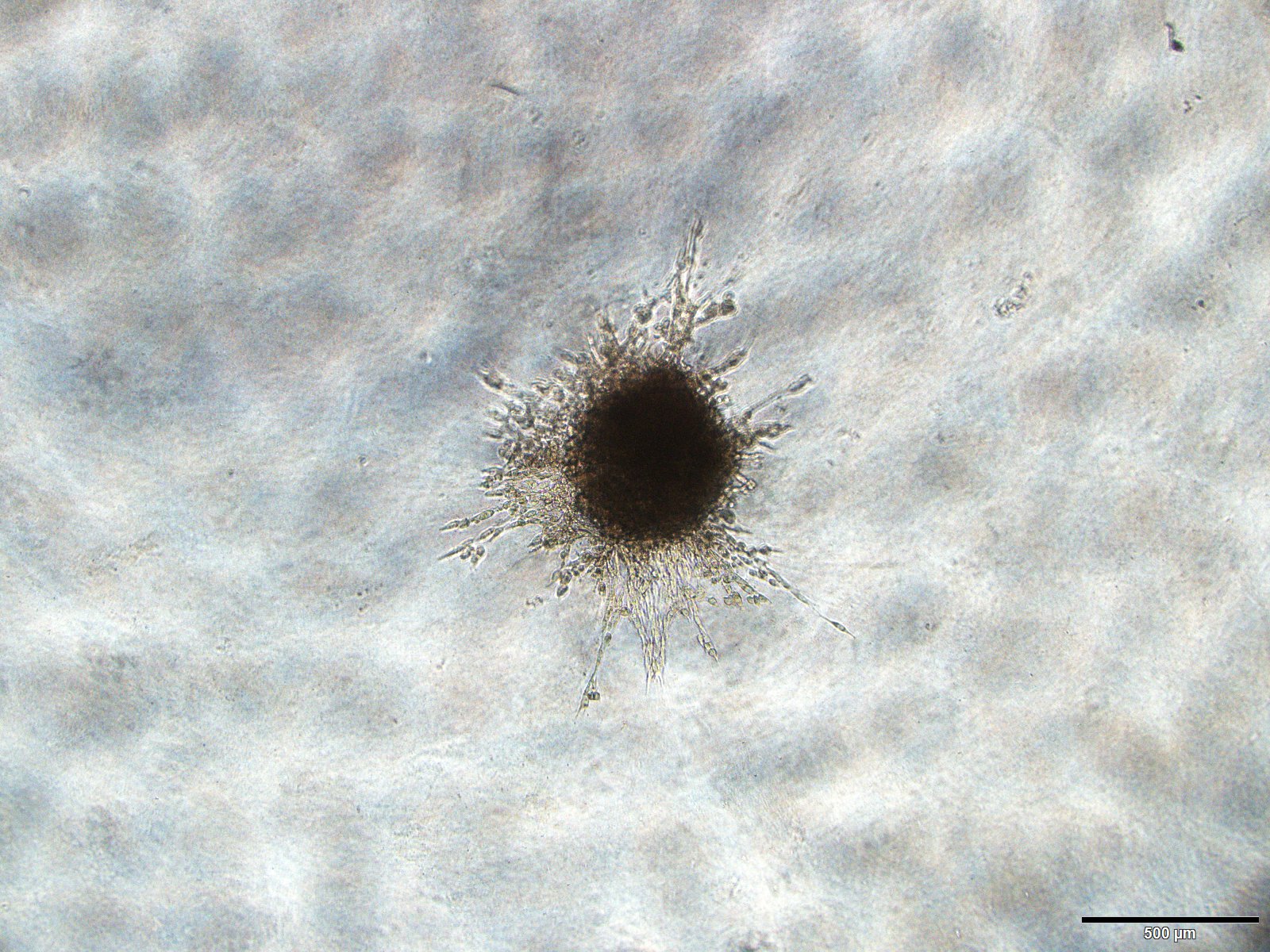

Supplement: Supplementary file 11 — Source Data [file 41467_2020_17139_MOESM11_ESM.zip › Source_Data/Figure 4g HS683 invasion images/HS683_Cont_1.jpg]

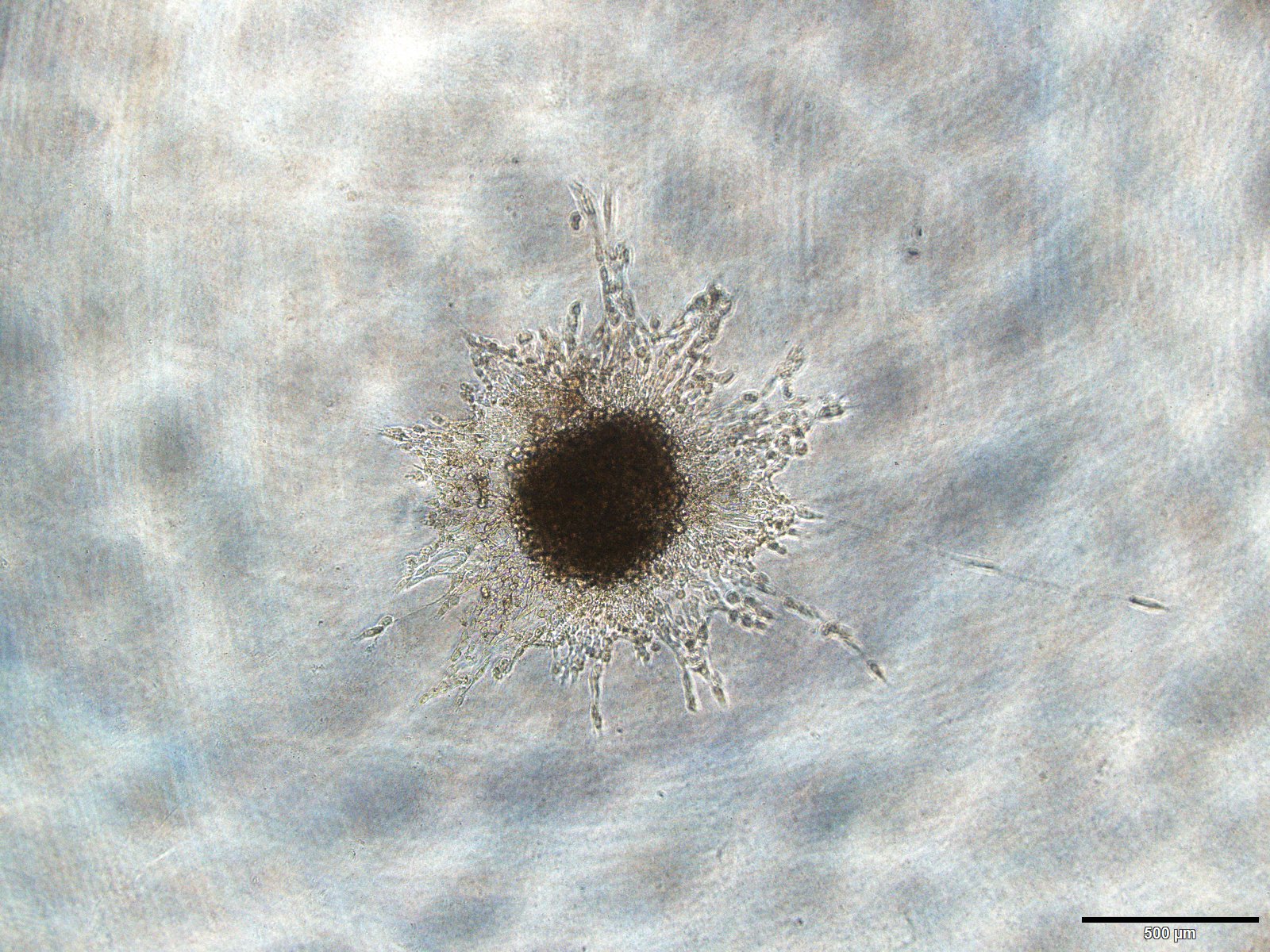

Supplement: Supplementary file 11 — Source Data [file 41467_2020_17139_MOESM11_ESM.zip › Source_Data/Figure 4g HS683 invasion images/HS683_Cont_2.jpg]

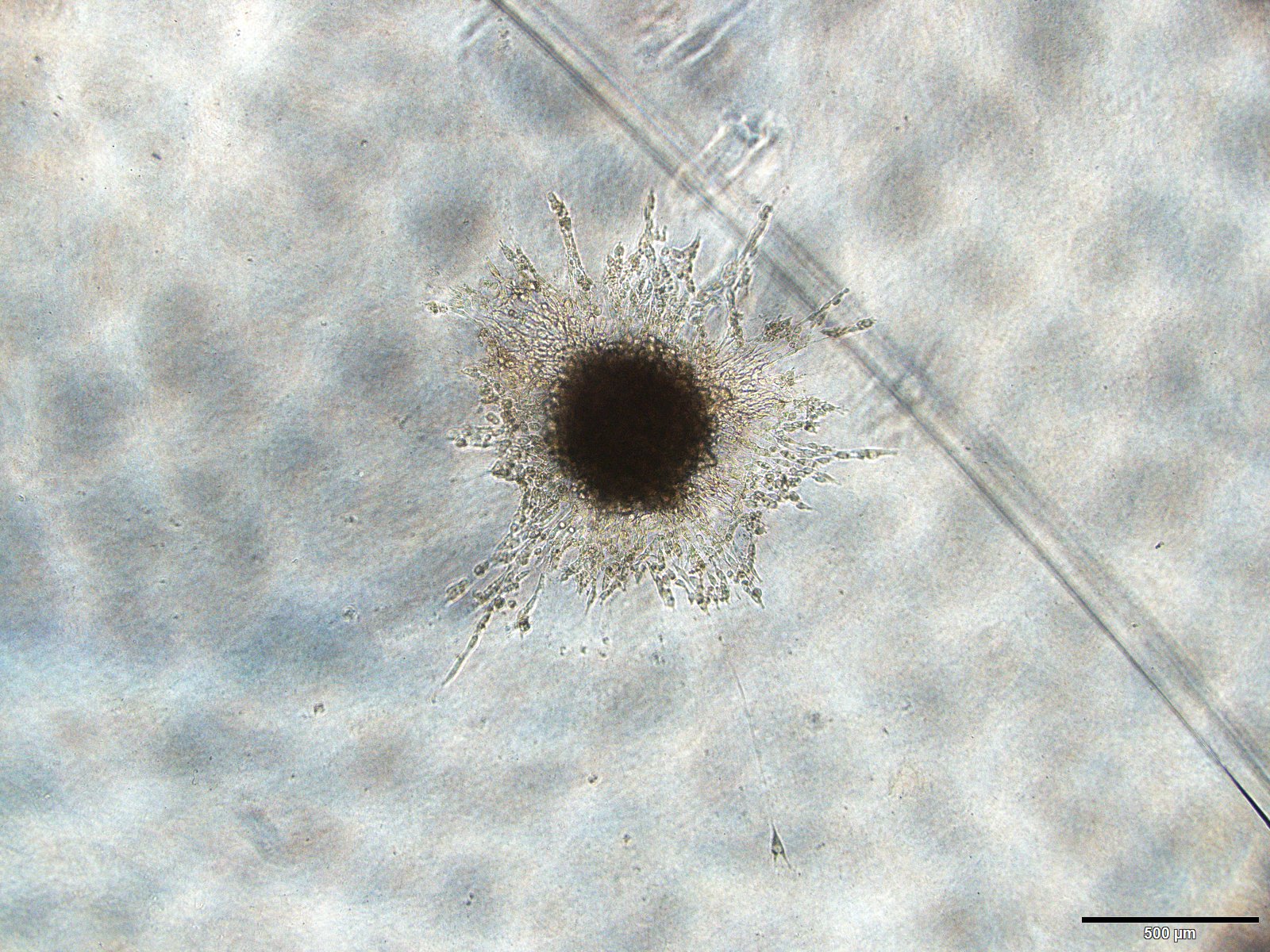

Supplement: Supplementary file 11 — Source Data [file 41467_2020_17139_MOESM11_ESM.zip › Source_Data/Figure 4g HS683 invasion images/HS683_Cont_3.jpg]

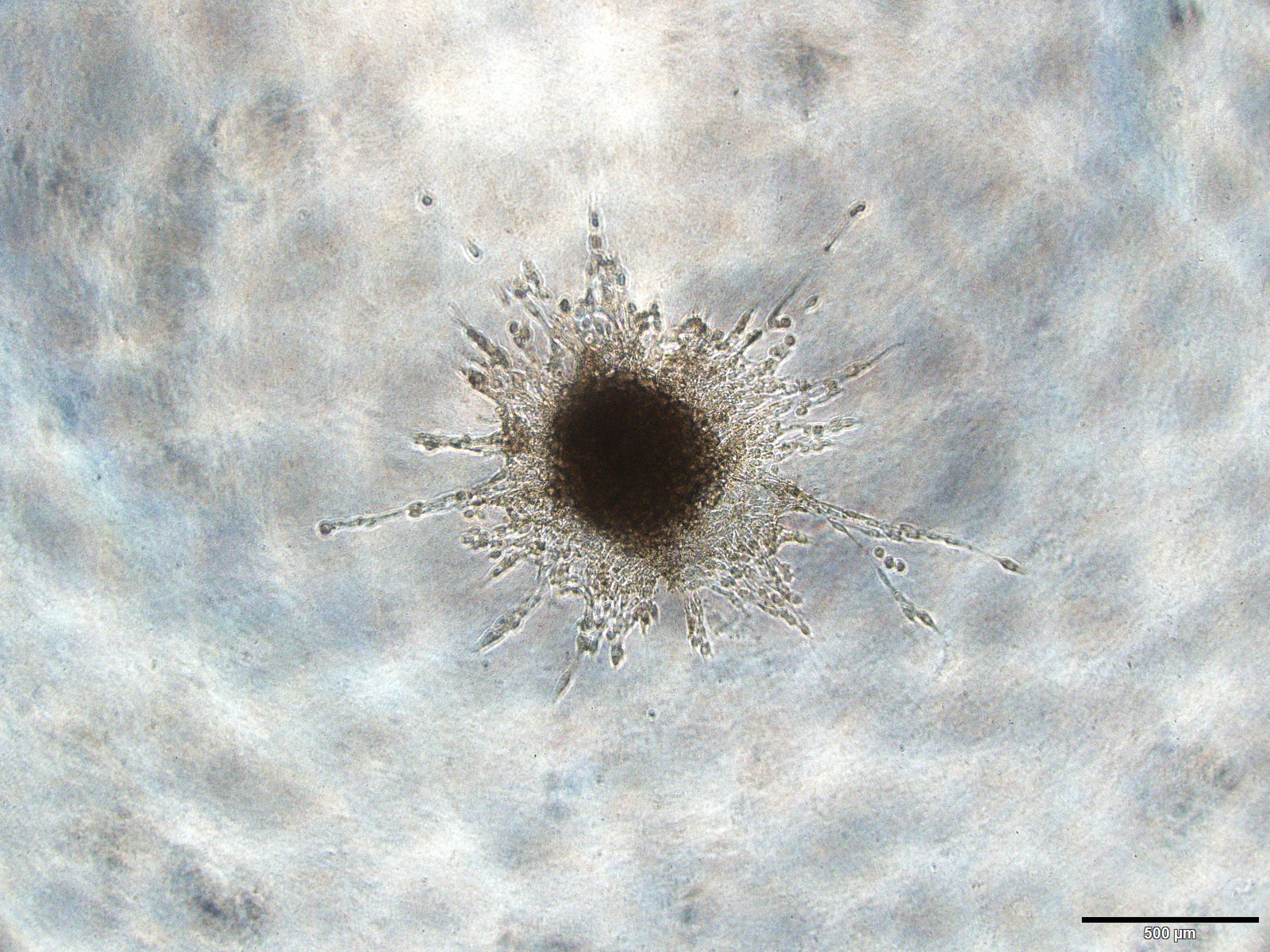

Supplement: Supplementary file 11 — Source Data [file 41467_2020_17139_MOESM11_ESM.zip › Source_Data/Figure 4g HS683 invasion images/HS683_NCT502_3.5uM_1.jpg]

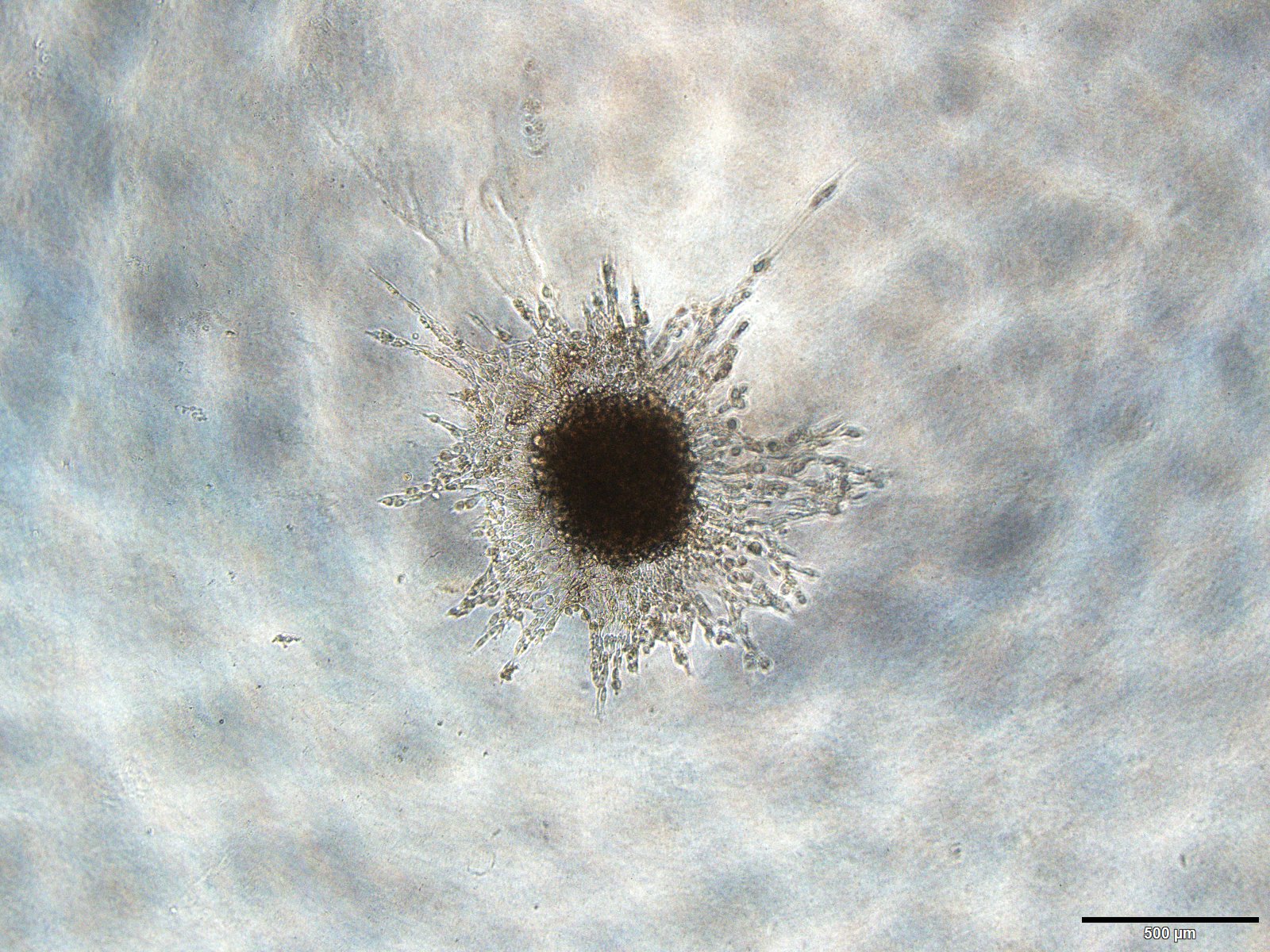

Supplement: Supplementary file 11 — Source Data [file 41467_2020_17139_MOESM11_ESM.zip › Source_Data/Figure 4g HS683 invasion images/HS683_NCT502_3.5uM_2.jpg]

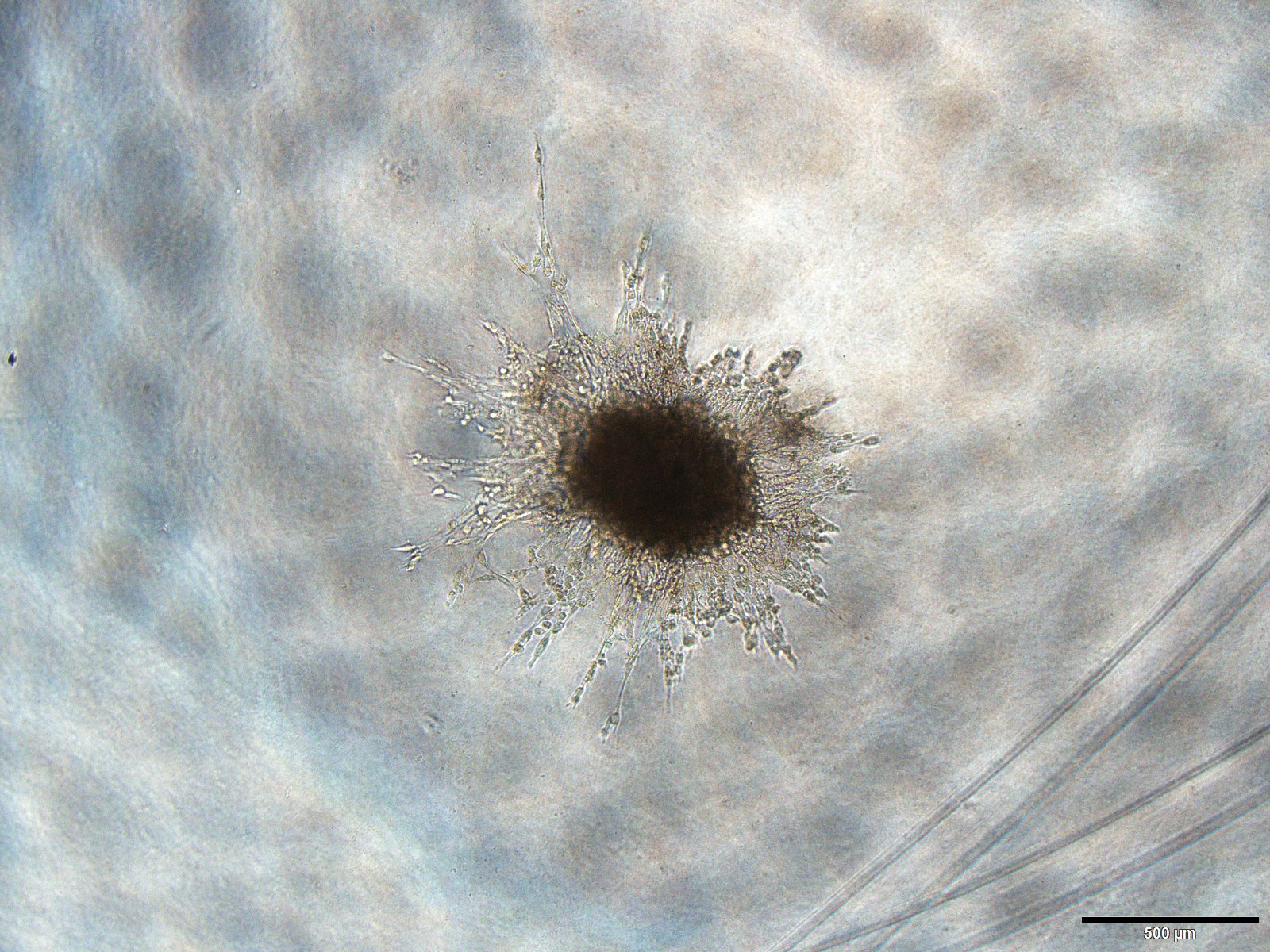

Supplement: Supplementary file 11 — Source Data [file 41467_2020_17139_MOESM11_ESM.zip › Source_Data/Figure 4g HS683 invasion images/HS683_NCT502_3.5uM_3.jpg]

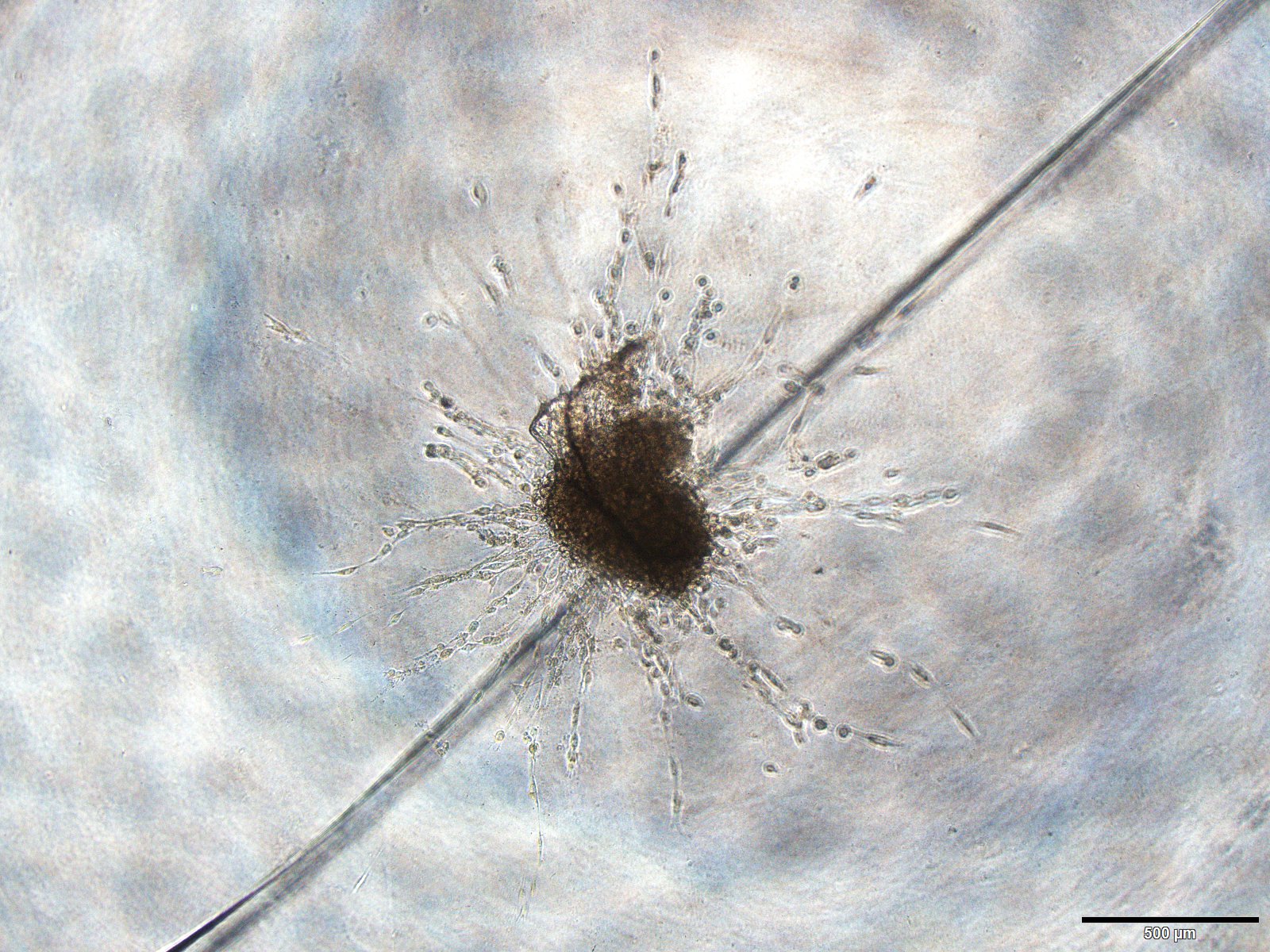

Supplement: Supplementary file 11 — Source Data [file 41467_2020_17139_MOESM11_ESM.zip › Source_Data/Figure 4g HS683 invasion images/HS683_NCT502_35uM_1.jpg]

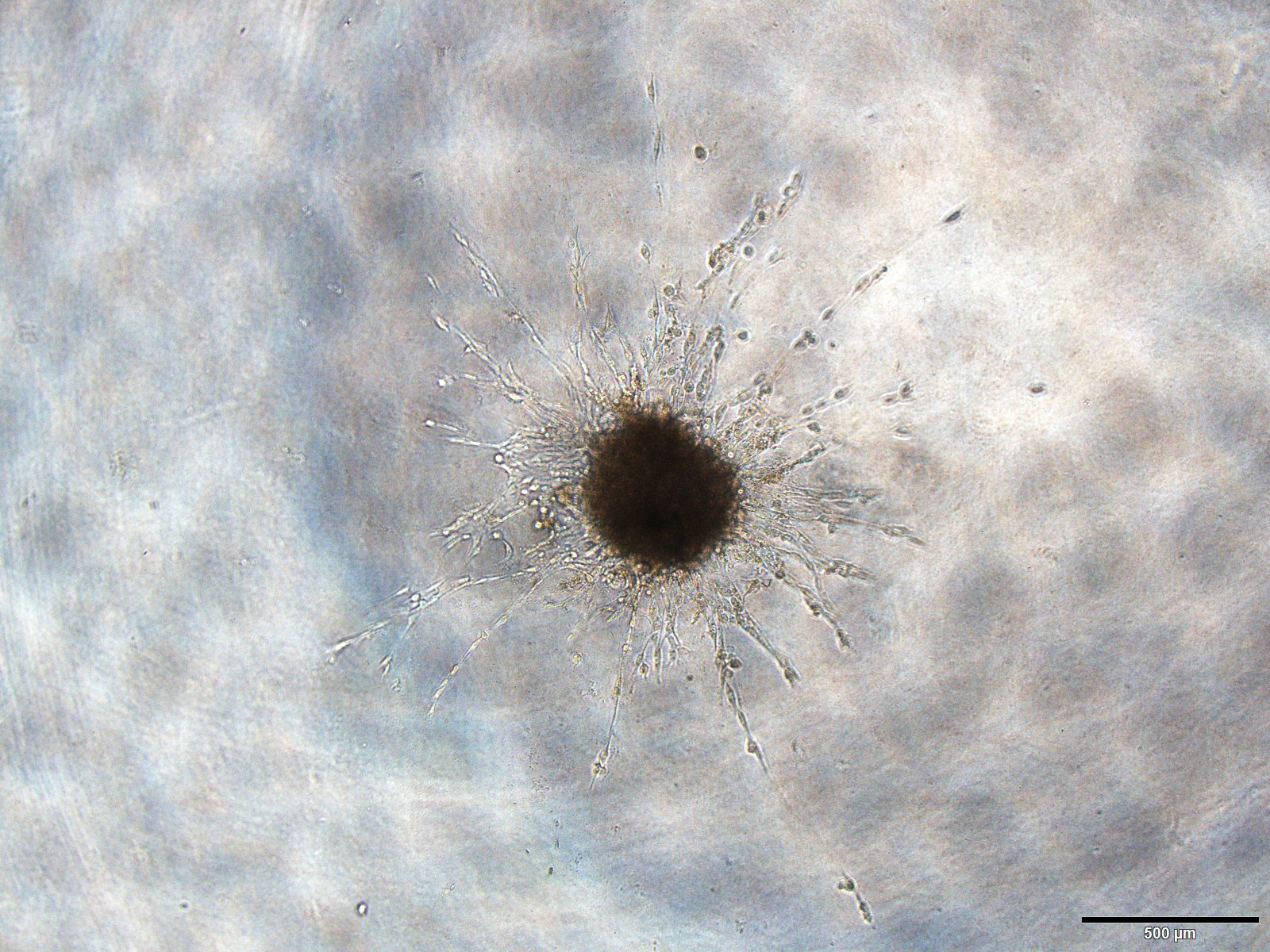

Supplement: Supplementary file 11 — Source Data [file 41467_2020_17139_MOESM11_ESM.zip › Source_Data/Figure 4g HS683 invasion images/HS683_NCT502_35uM_2.jpg]

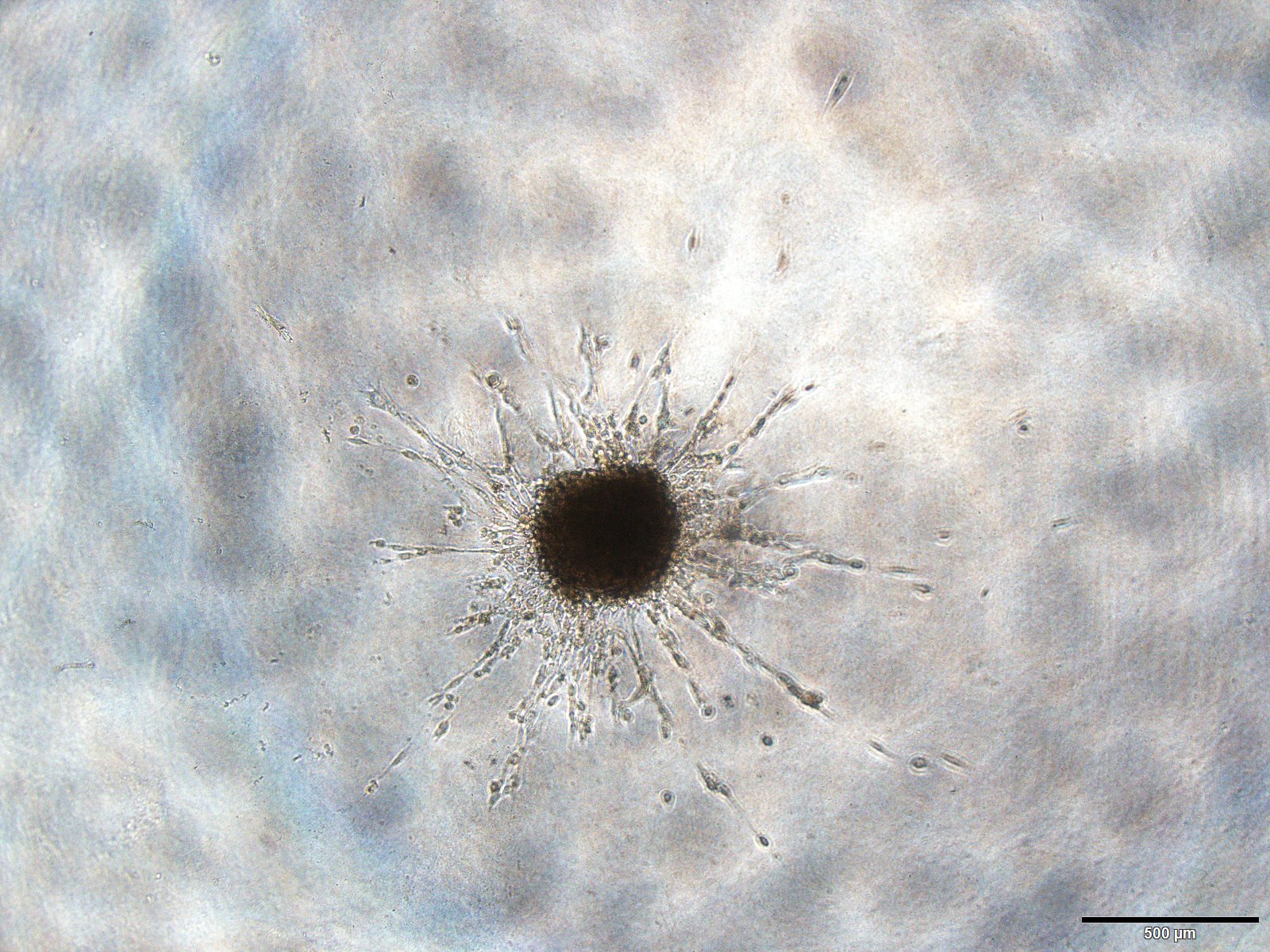

Supplement: Supplementary file 11 — Source Data [file 41467_2020_17139_MOESM11_ESM.zip › Source_Data/Figure 4g HS683 invasion images/HS683_NCT502_35uM_3.jpg]

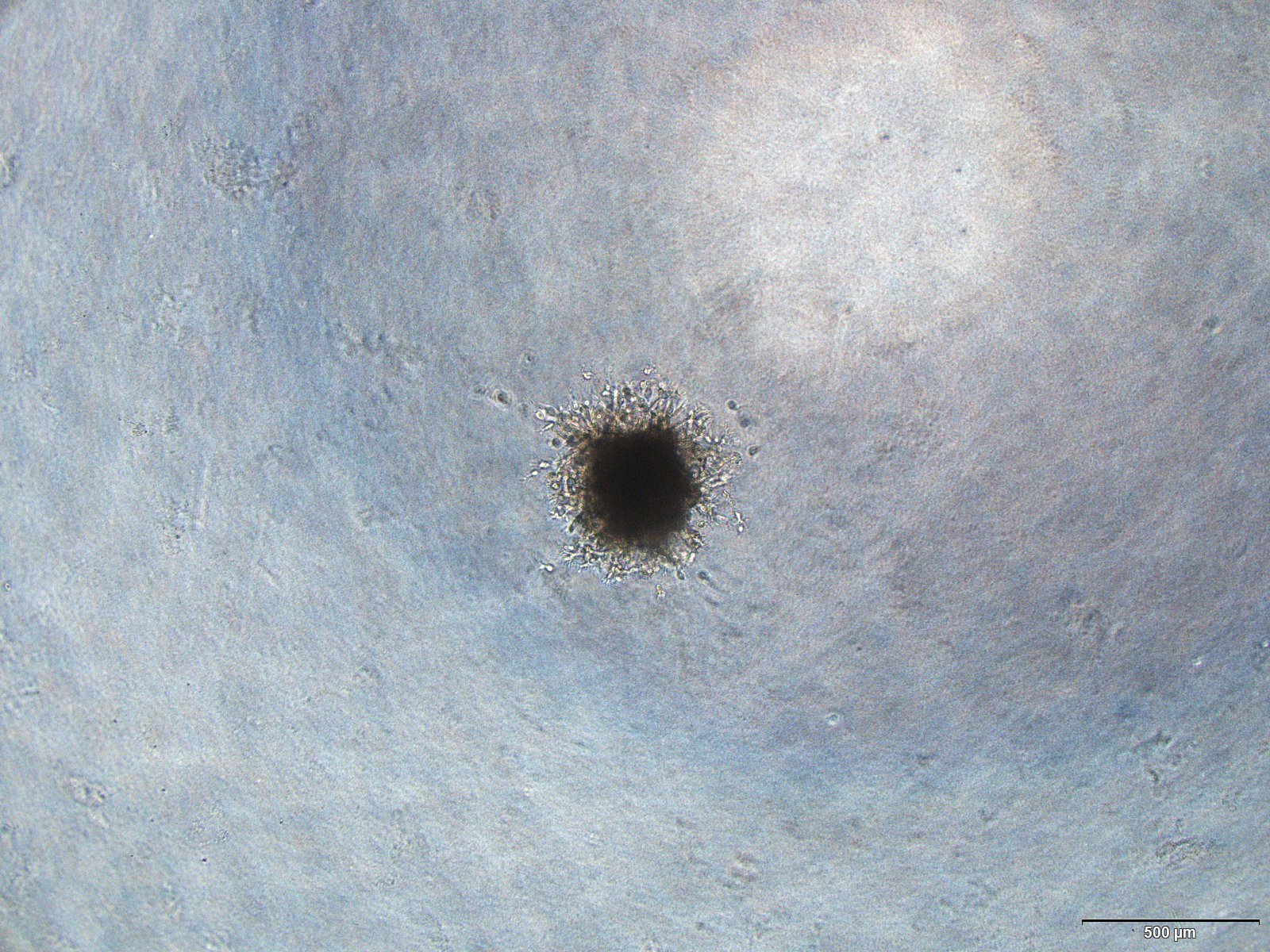

Supplement: Supplementary file 11 — Source Data [file 41467_2020_17139_MOESM11_ESM.zip › Source_Data/Figure 4g SNU1105 invasion images/SNU1105_Cont_1.jpg]

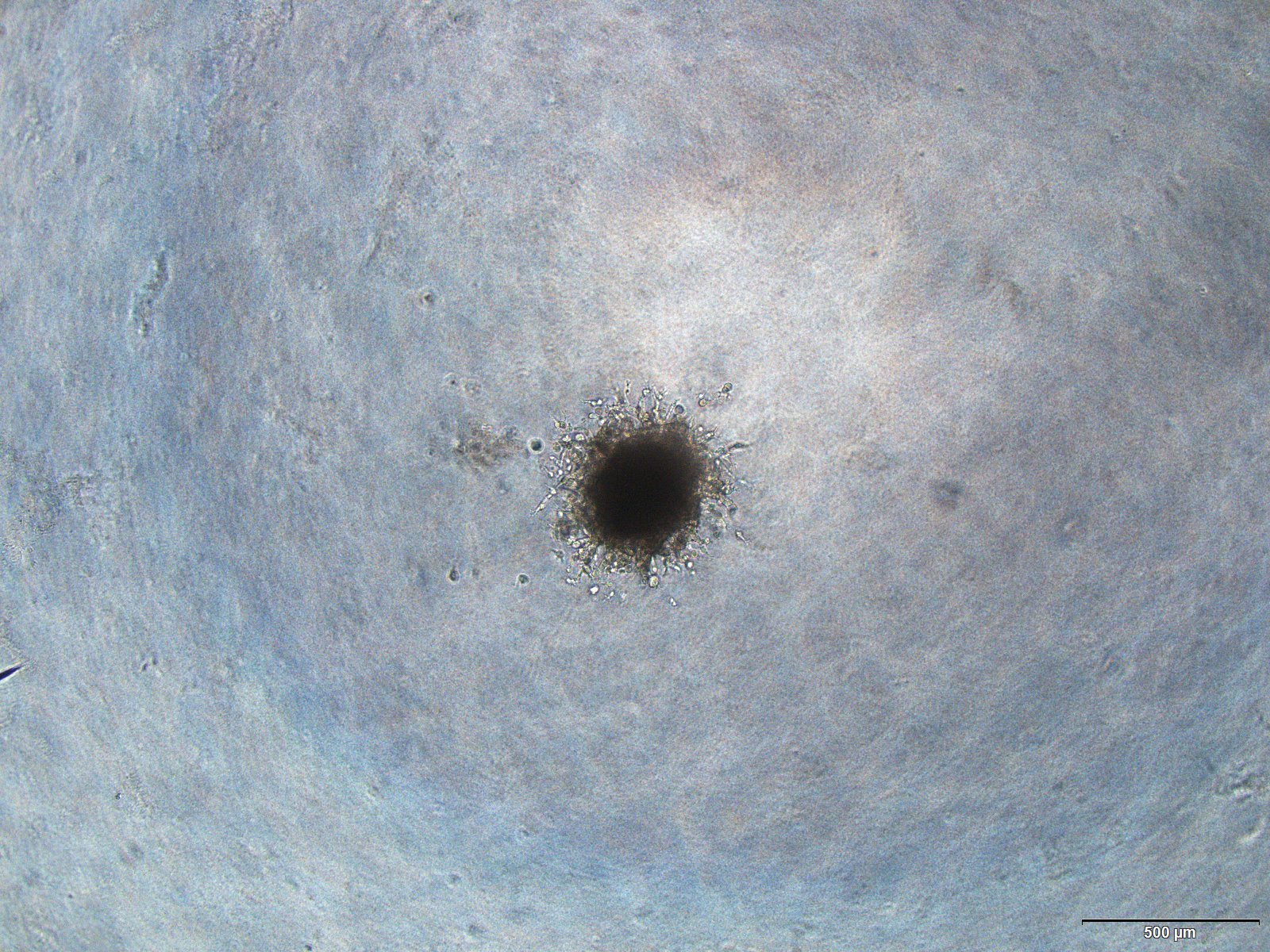

Supplement: Supplementary file 11 — Source Data [file 41467_2020_17139_MOESM11_ESM.zip › Source_Data/Figure 4g SNU1105 invasion images/SNU1105_Cont_2.jpg]

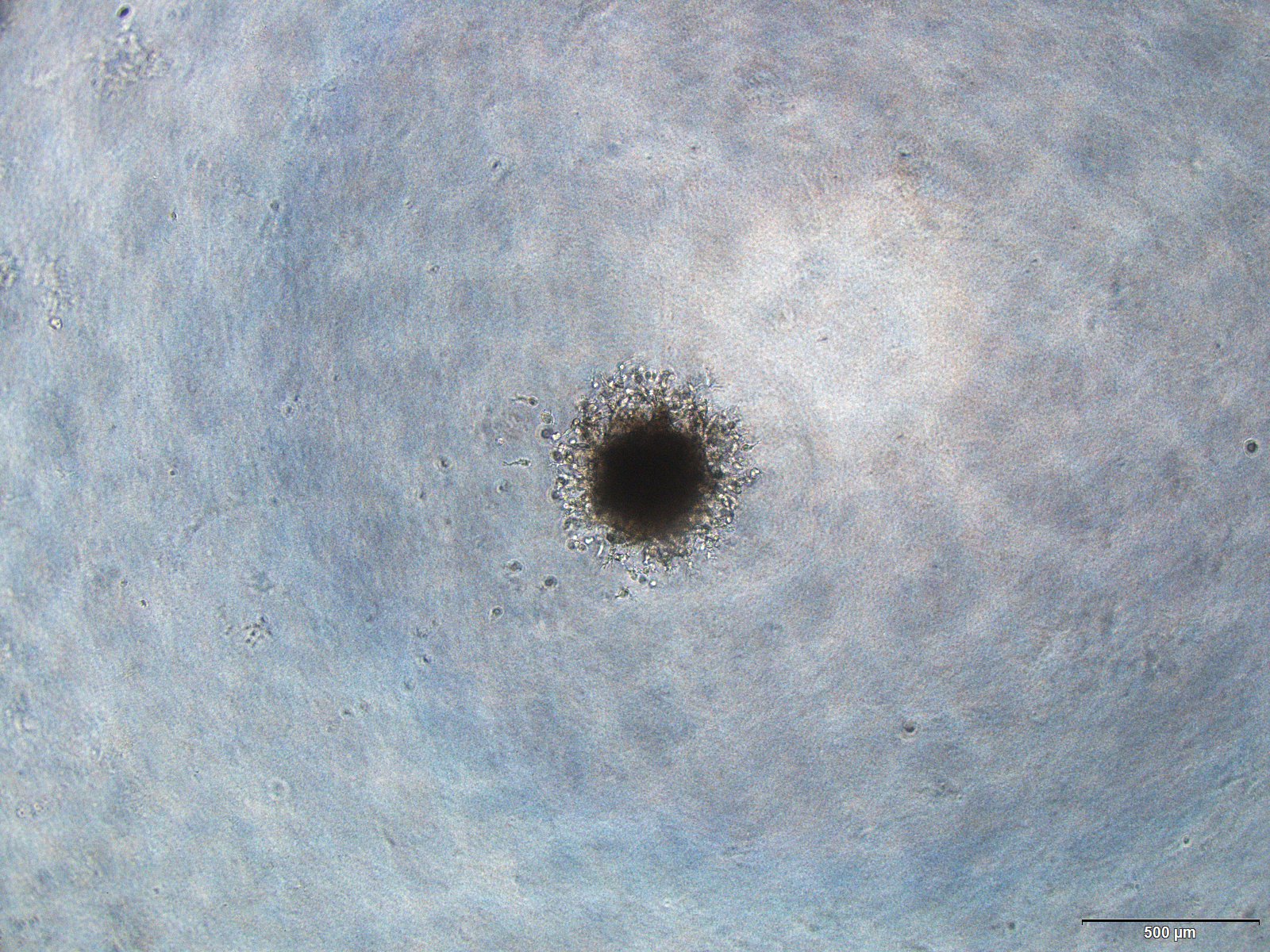

Supplement: Supplementary file 11 — Source Data [file 41467_2020_17139_MOESM11_ESM.zip › Source_Data/Figure 4g SNU1105 invasion images/SNU1105_Cont_3.jpg]

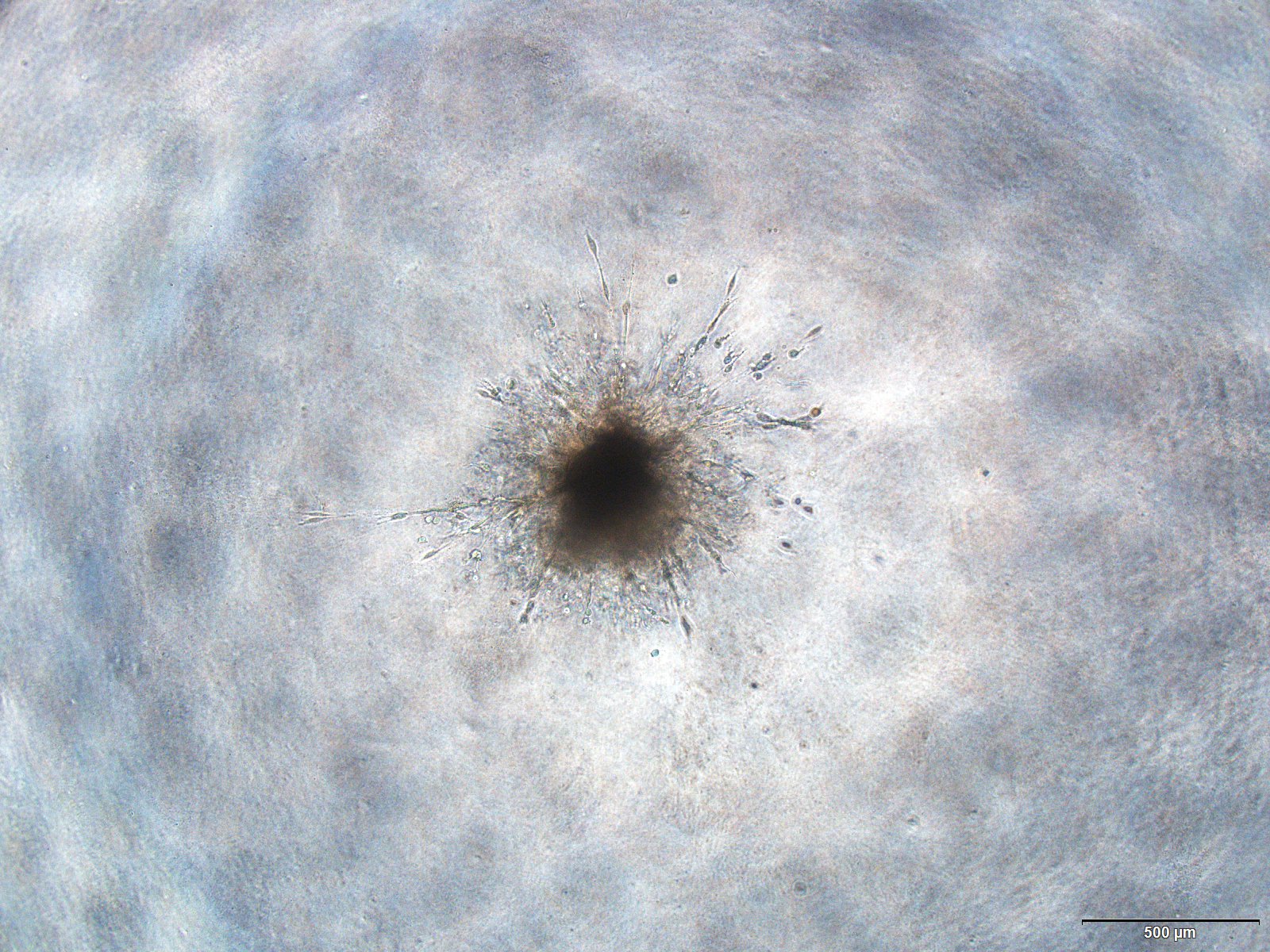

Supplement: Supplementary file 11 — Source Data [file 41467_2020_17139_MOESM11_ESM.zip › Source_Data/Figure 4g SNU1105 invasion images/SNU1105_NCT502_3.5uM_1.jpg]

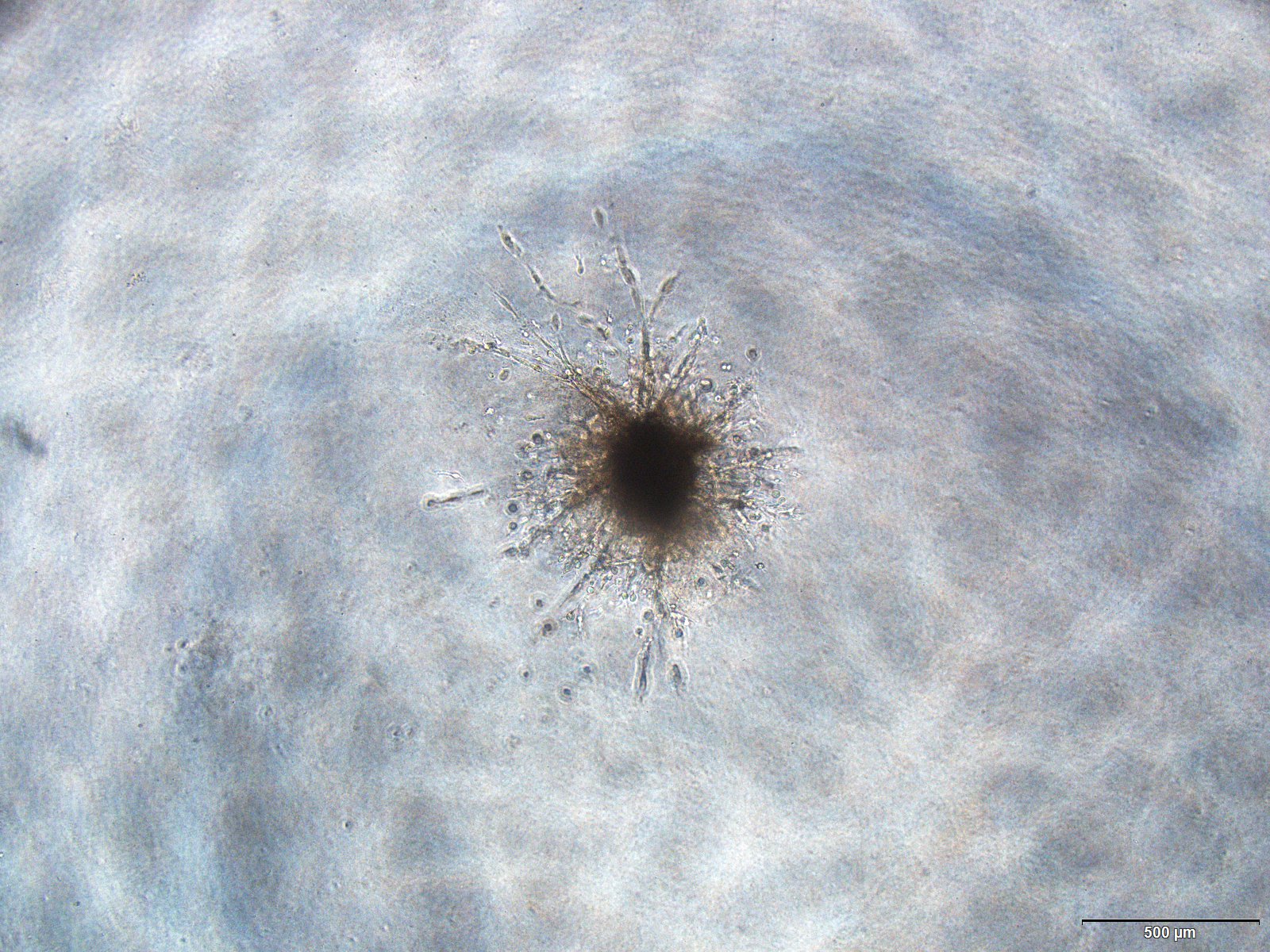

Supplement: Supplementary file 11 — Source Data [file 41467_2020_17139_MOESM11_ESM.zip › Source_Data/Figure 4g SNU1105 invasion images/SNU1105_NCT502_3.5uM_2.jpg]

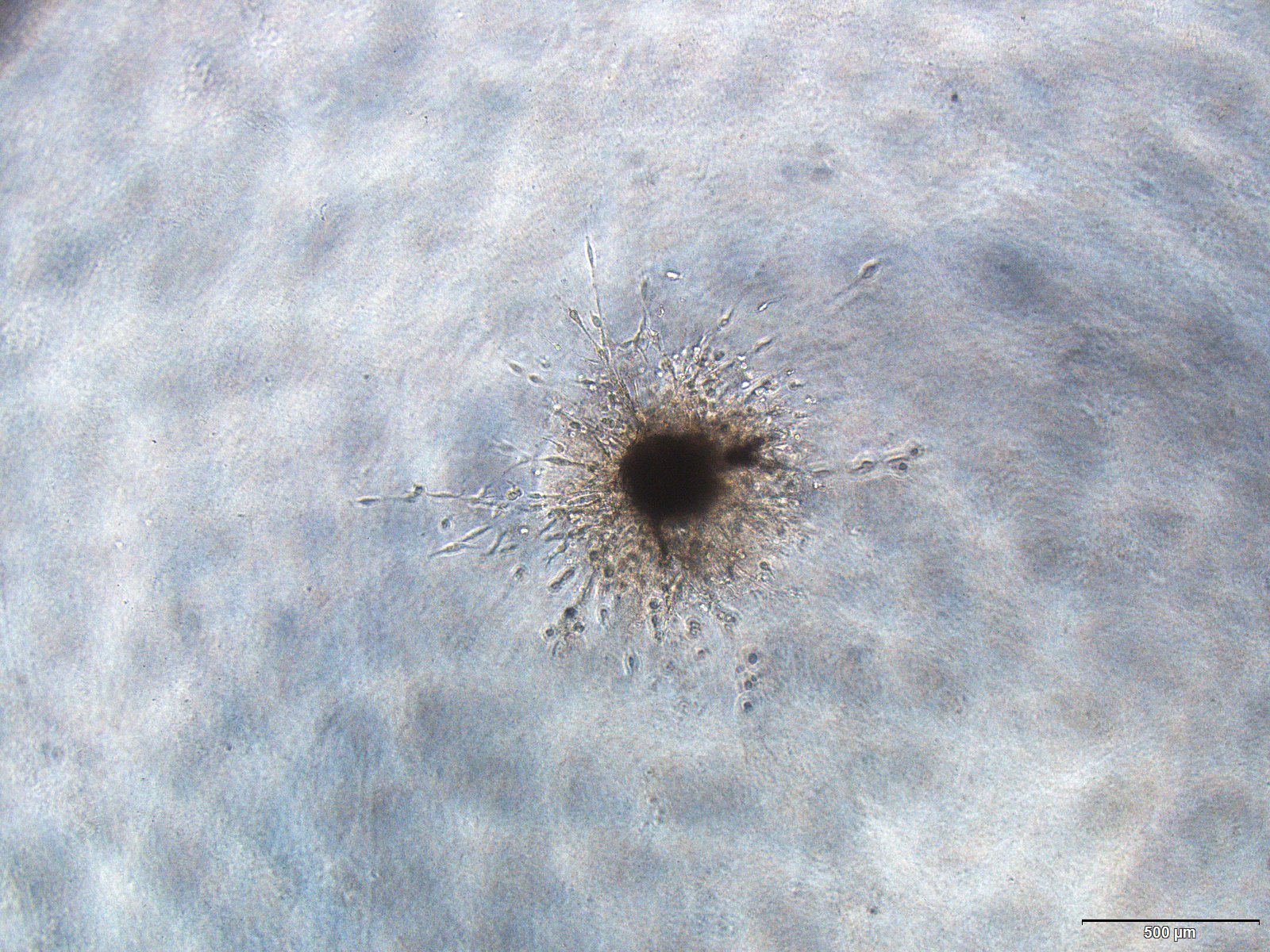

Supplement: Supplementary file 11 — Source Data [file 41467_2020_17139_MOESM11_ESM.zip › Source_Data/Figure 4g SNU1105 invasion images/SNU1105_NCT502_3.5uM_3.jpg]

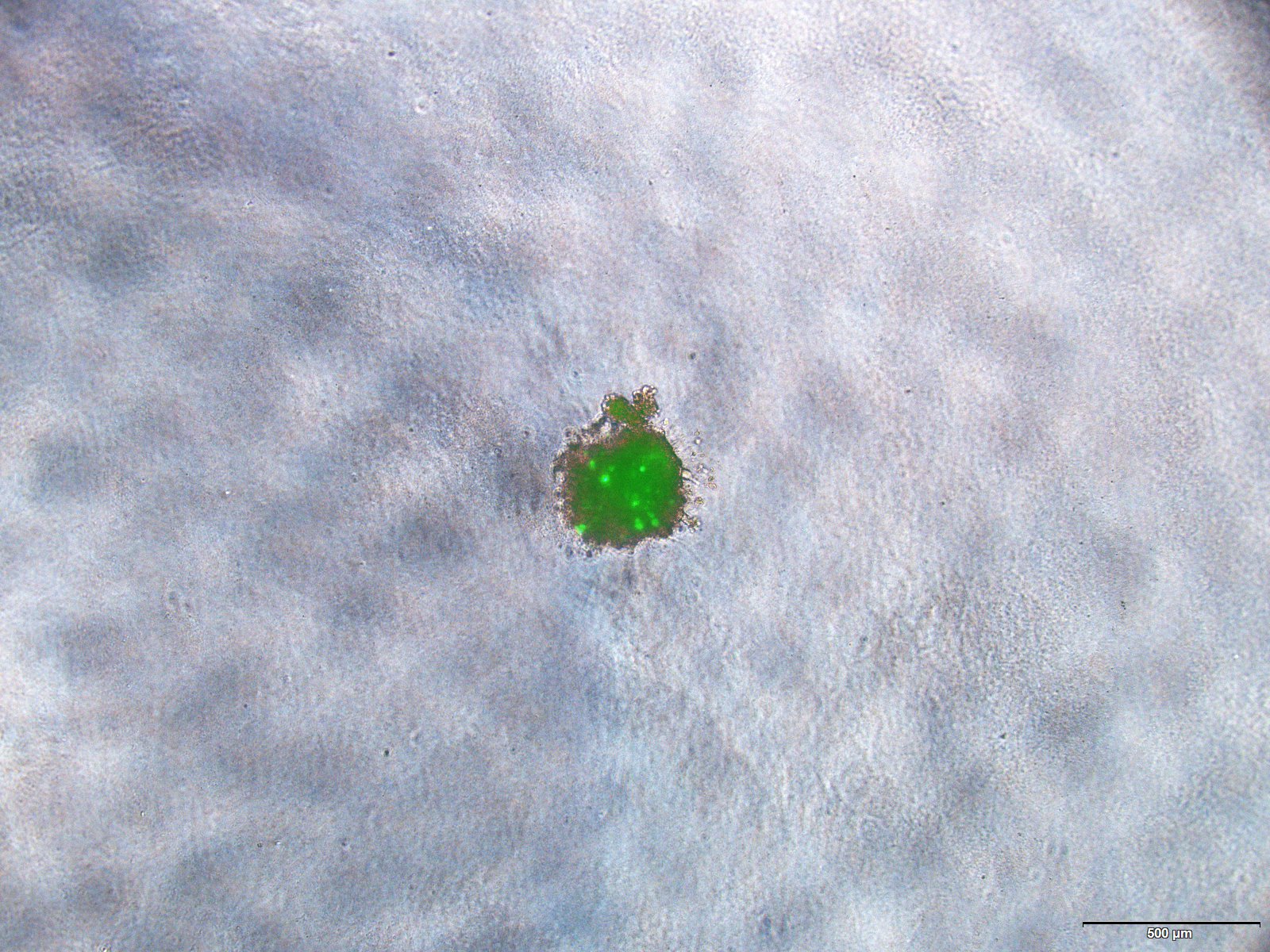

Supplement: Supplementary file 11 — Source Data [file 41467_2020_17139_MOESM11_ESM.zip › Source_Data/Supplementary Figure 4e KNS81 invasion images/KNS81_PHGDH_1.jpg]

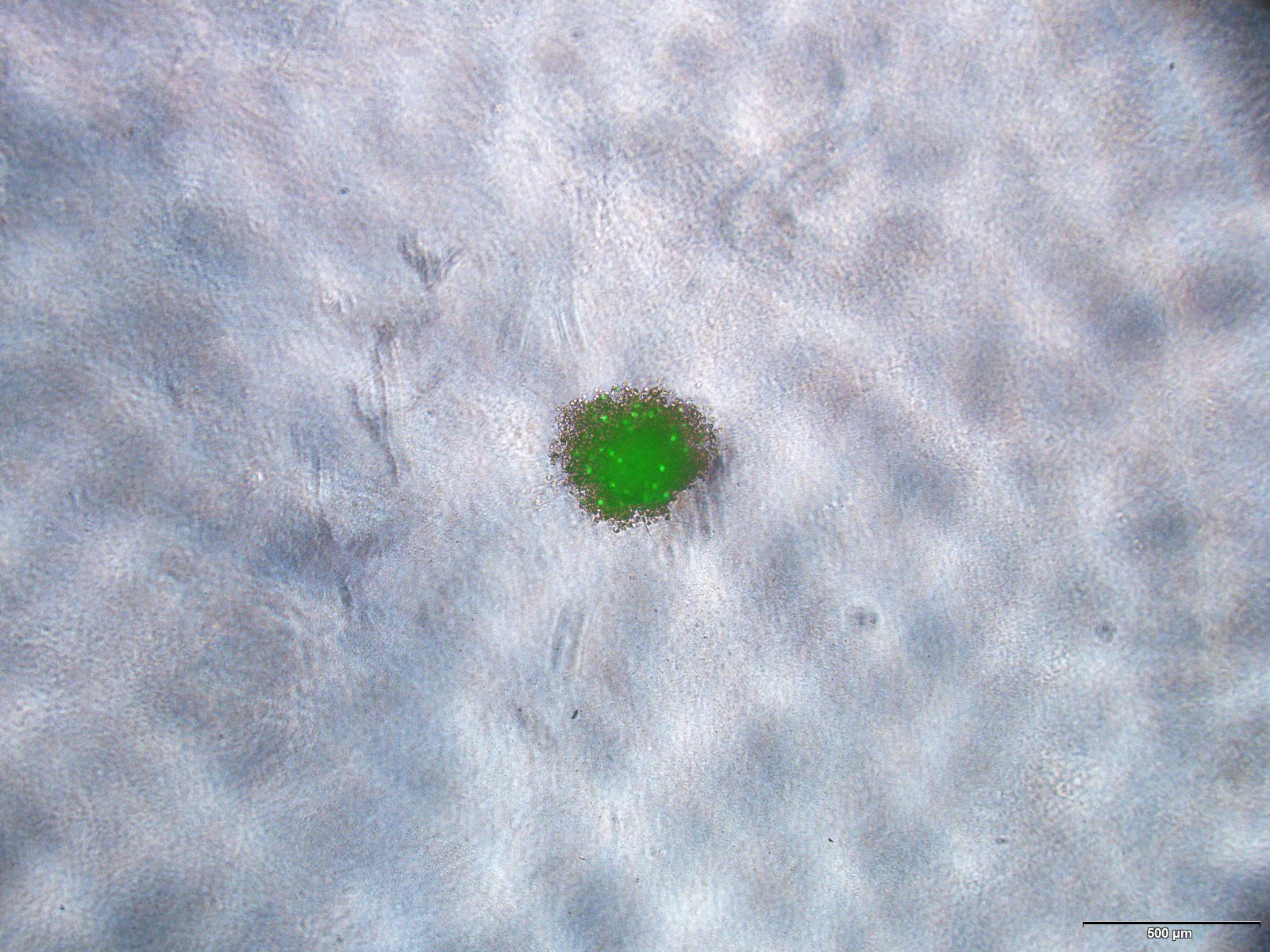

Supplement: Supplementary file 11 — Source Data [file 41467_2020_17139_MOESM11_ESM.zip › Source_Data/Supplementary Figure 4e KNS81 invasion images/KNS81_PHGDH_2.jpg]

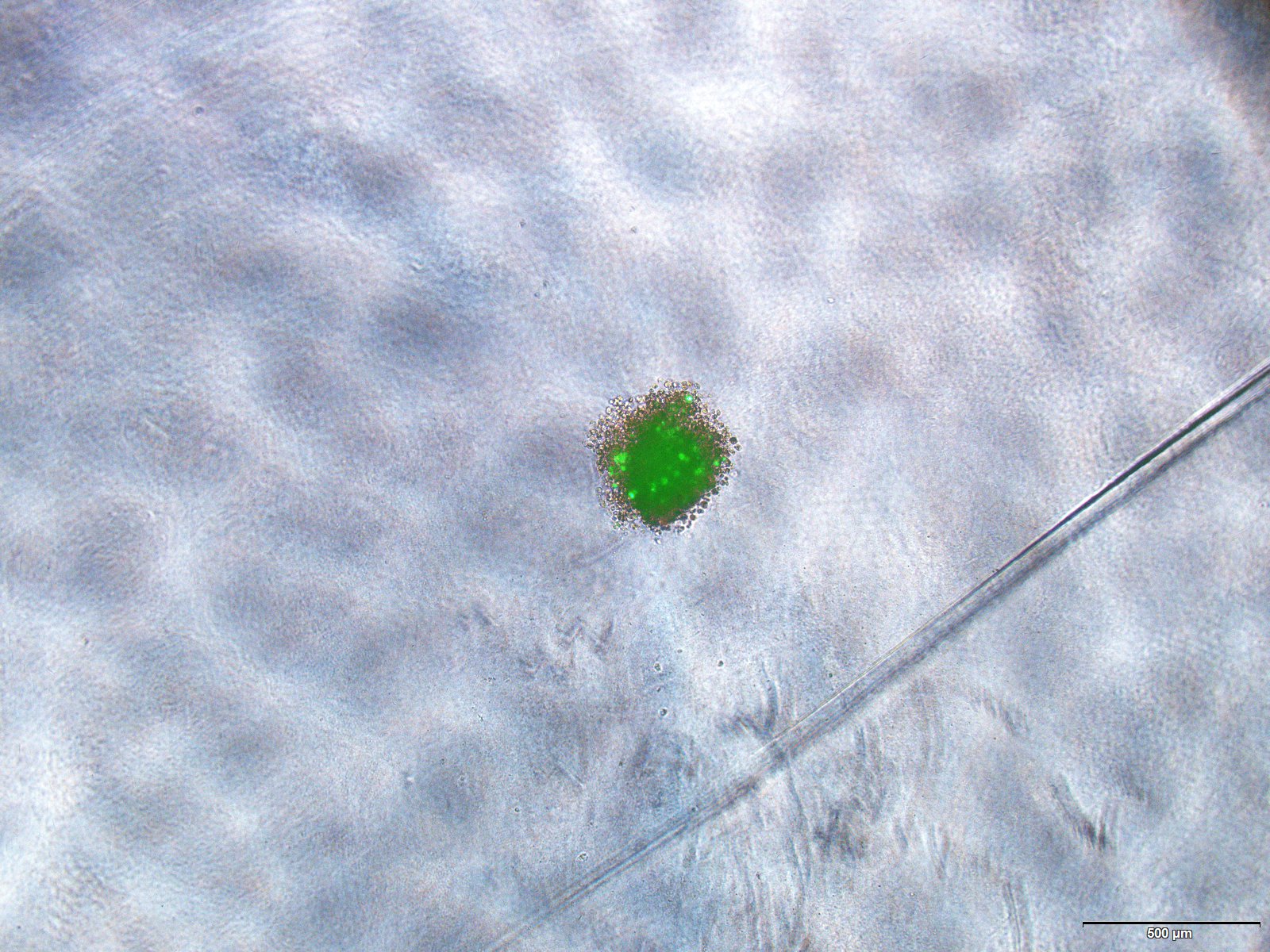

Supplement: Supplementary file 11 — Source Data [file 41467_2020_17139_MOESM11_ESM.zip › Source_Data/Supplementary Figure 4e KNS81 invasion images/KNS81_PHGDH_3.jpg]

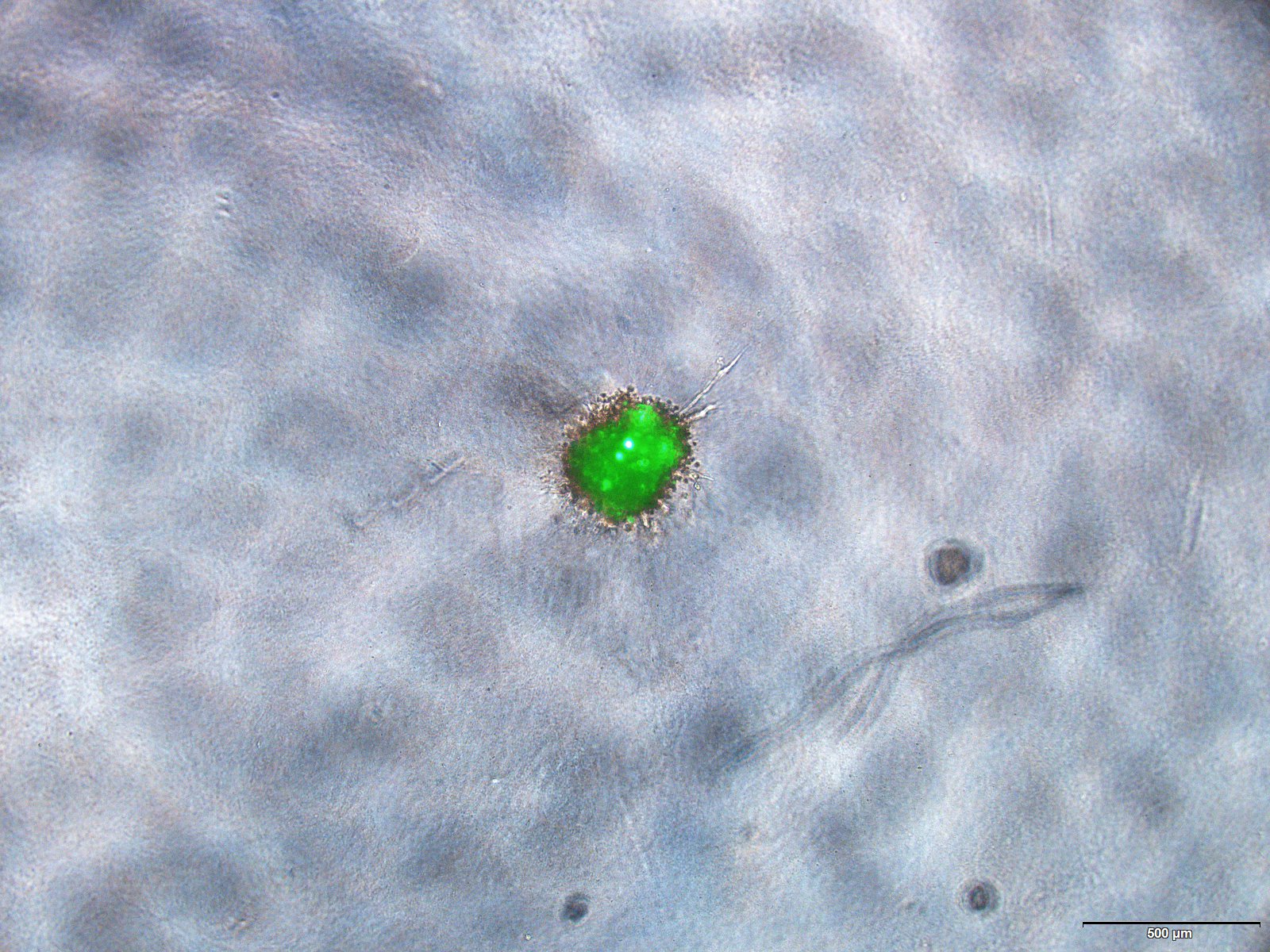

Supplement: Supplementary file 11 — Source Data [file 41467_2020_17139_MOESM11_ESM.zip › Source_Data/Supplementary Figure 4e KNS81 invasion images/KNS81_Vector_1.jpg]

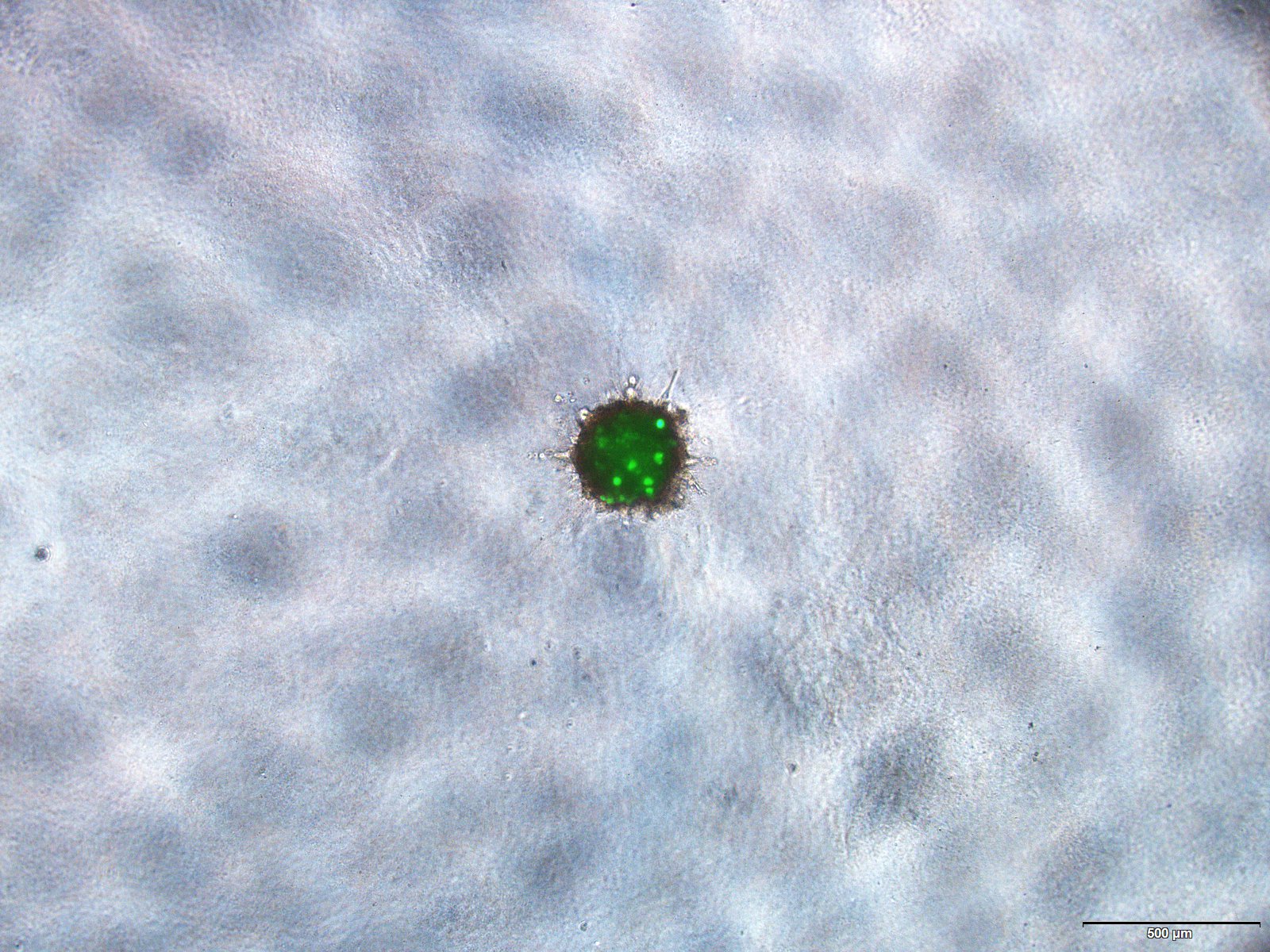

Supplement: Supplementary file 11 — Source Data [file 41467_2020_17139_MOESM11_ESM.zip › Source_Data/Supplementary Figure 4e KNS81 invasion images/KNS81_Vector_2.jpg]

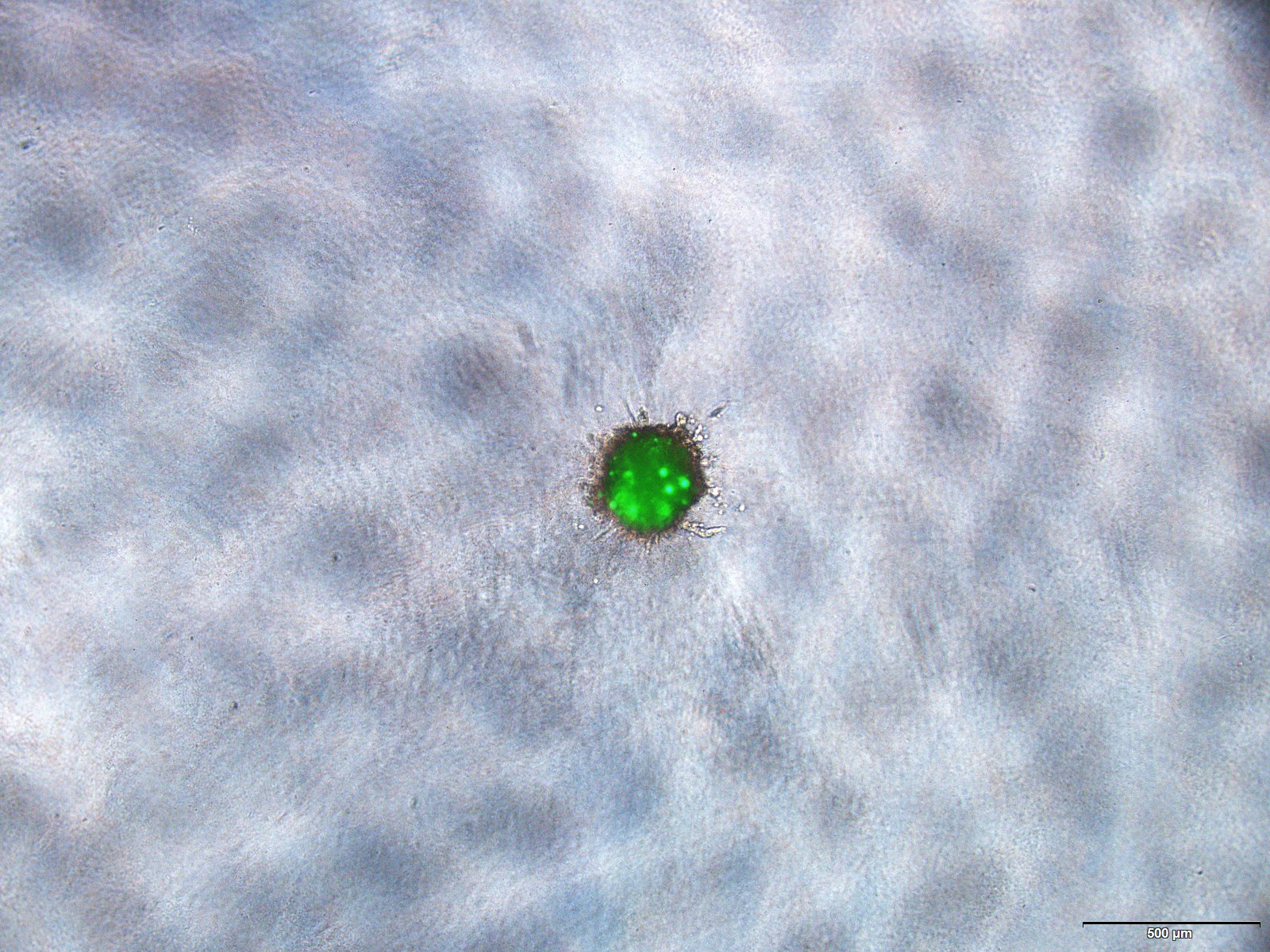

Supplement: Supplementary file 11 — Source Data [file 41467_2020_17139_MOESM11_ESM.zip › Source_Data/Supplementary Figure 4e KNS81 invasion images/KNS81_Vector_3.jpg]

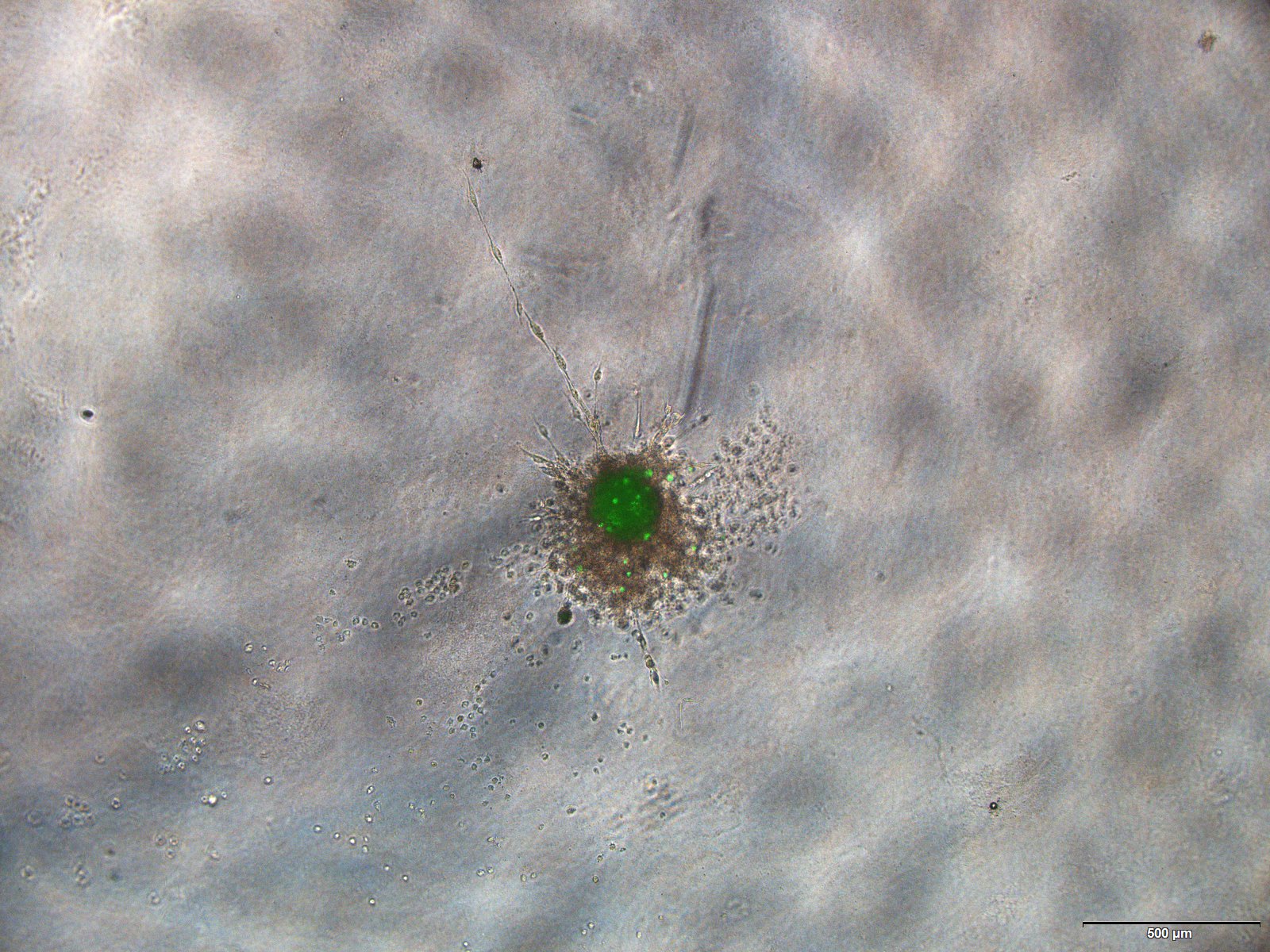

Supplement: Supplementary file 11 — Source Data [file 41467_2020_17139_MOESM11_ESM.zip › Source_Data/Supplementary Figure 4e SNU201 invasion images/SNU201_PHGDH_1.jpg]

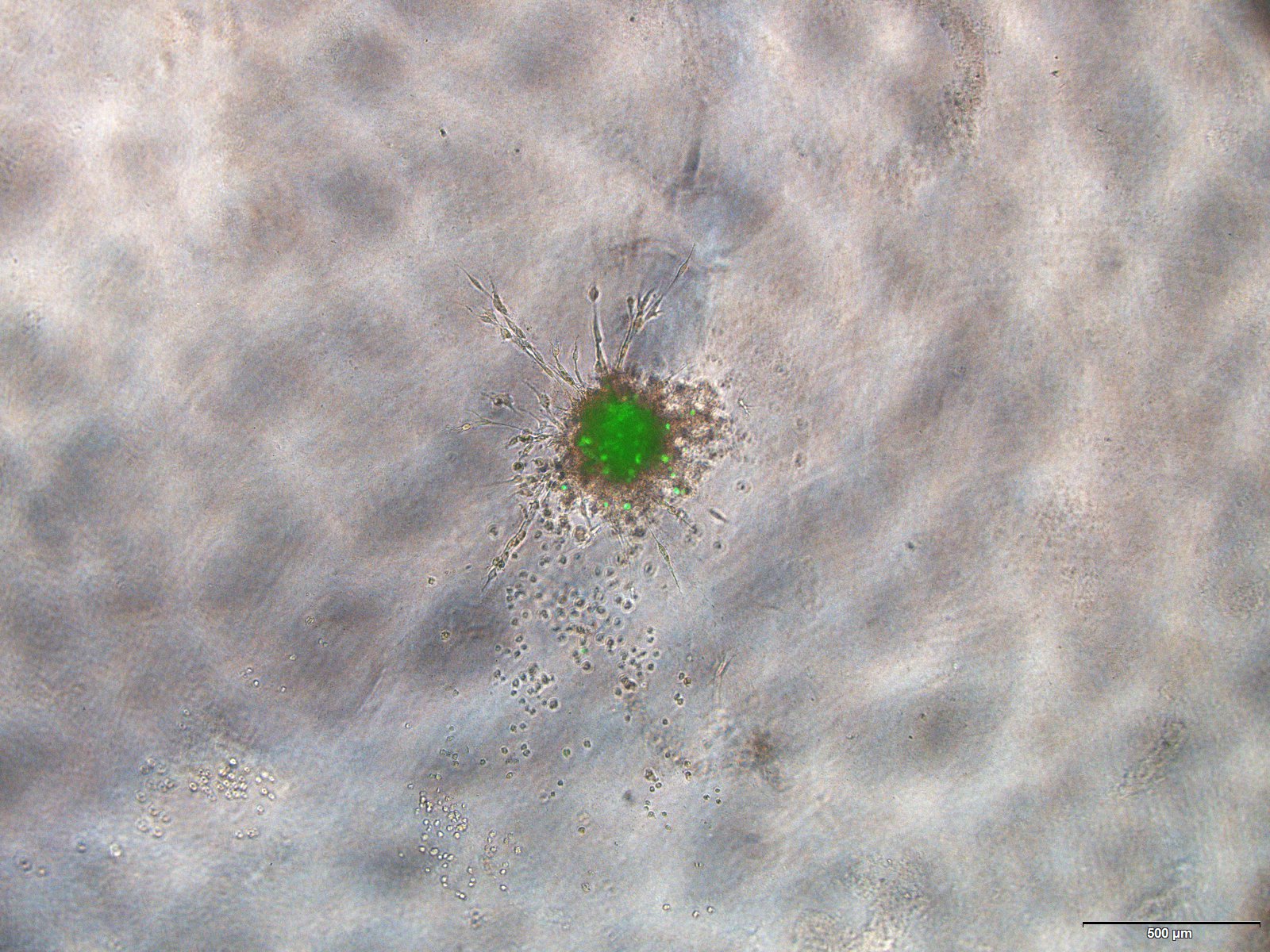

Supplement: Supplementary file 11 — Source Data [file 41467_2020_17139_MOESM11_ESM.zip › Source_Data/Supplementary Figure 4e SNU201 invasion images/SNU201_PHGDH_2.jpg]

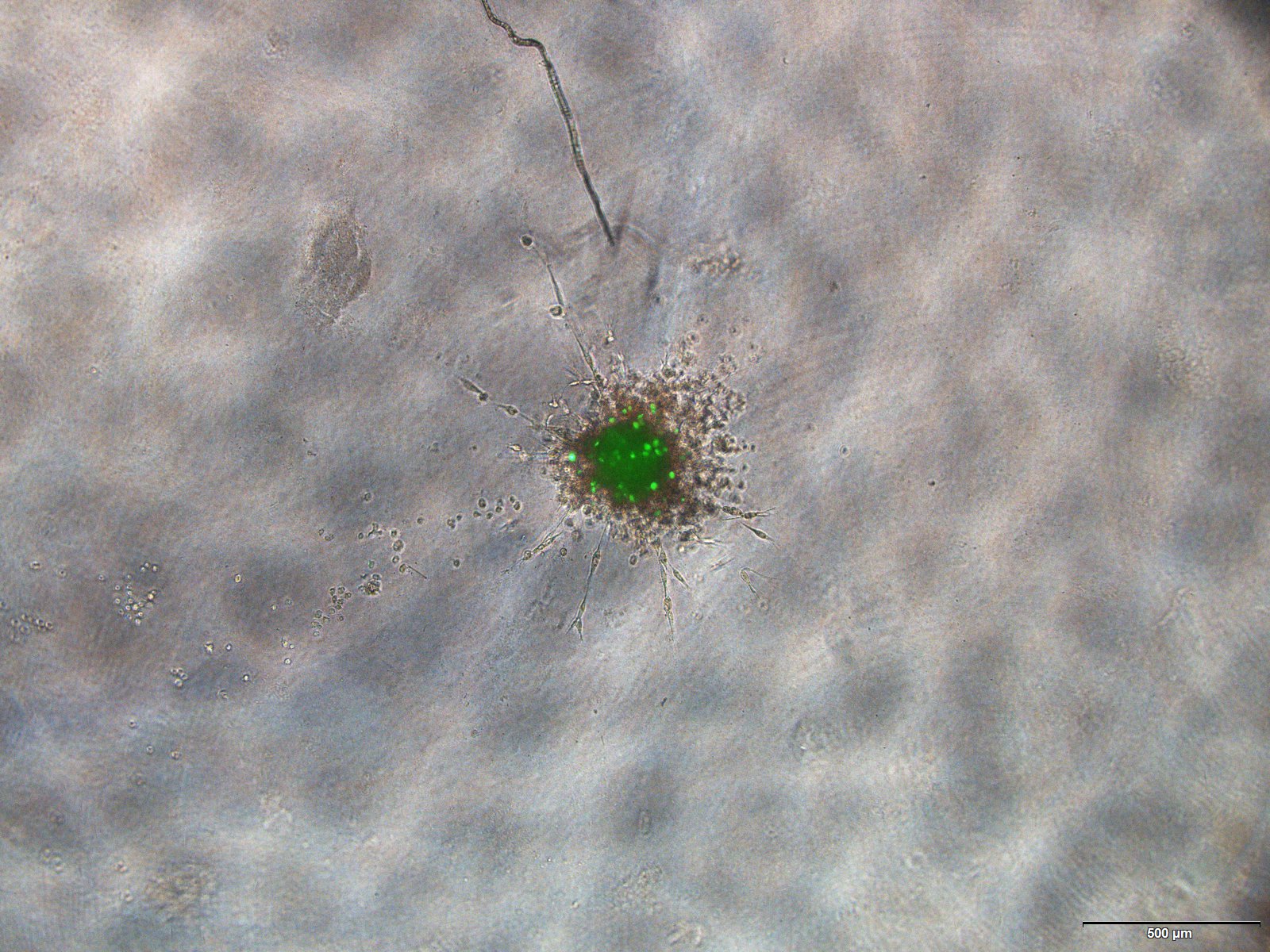

Supplement: Supplementary file 11 — Source Data [file 41467_2020_17139_MOESM11_ESM.zip › Source_Data/Supplementary Figure 4e SNU201 invasion images/SNU201_PHGDH_3.jpg]

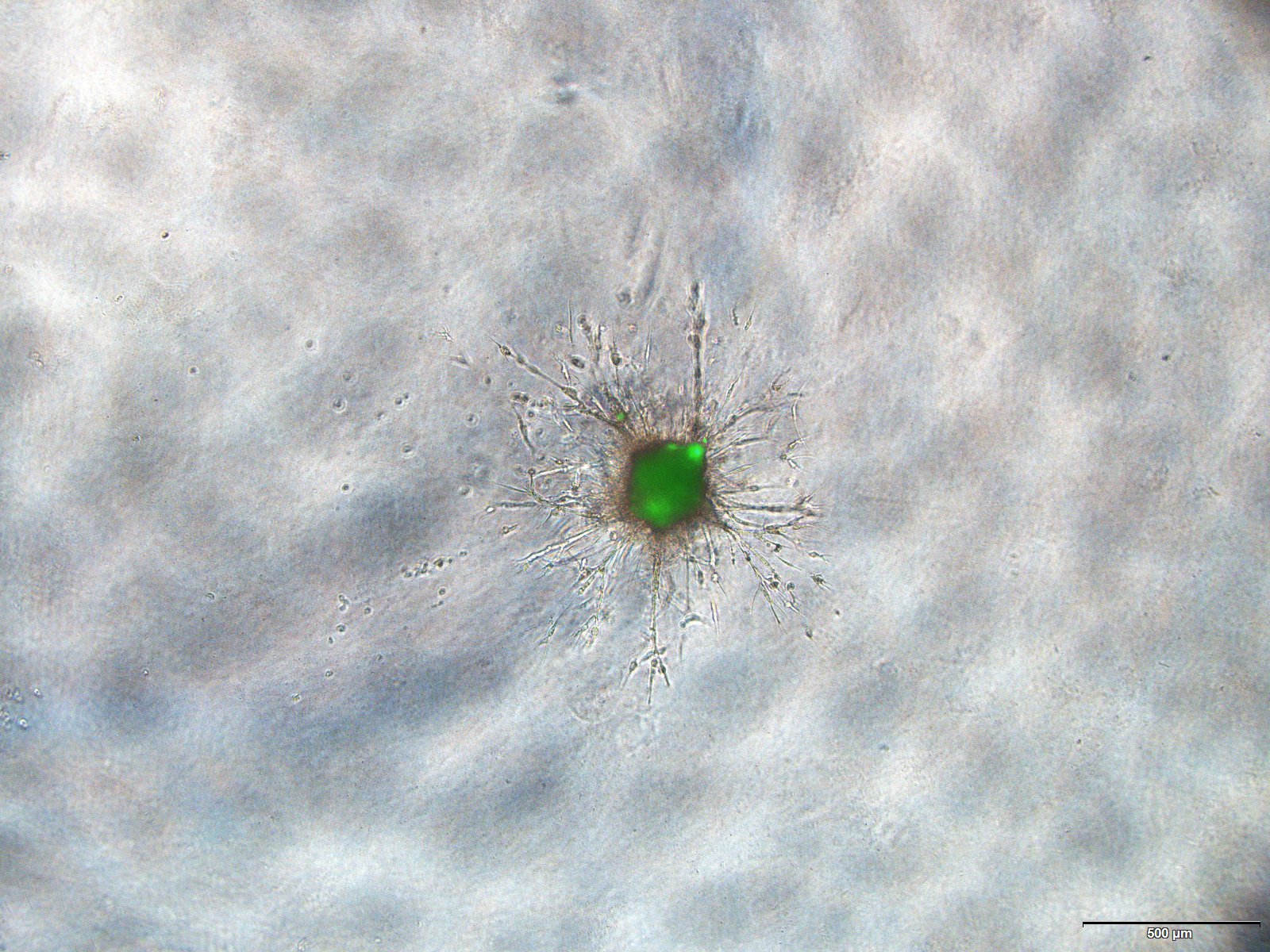

Supplement: Supplementary file 11 — Source Data [file 41467_2020_17139_MOESM11_ESM.zip › Source_Data/Supplementary Figure 4e SNU201 invasion images/SNU201_Vector_1.jpg]

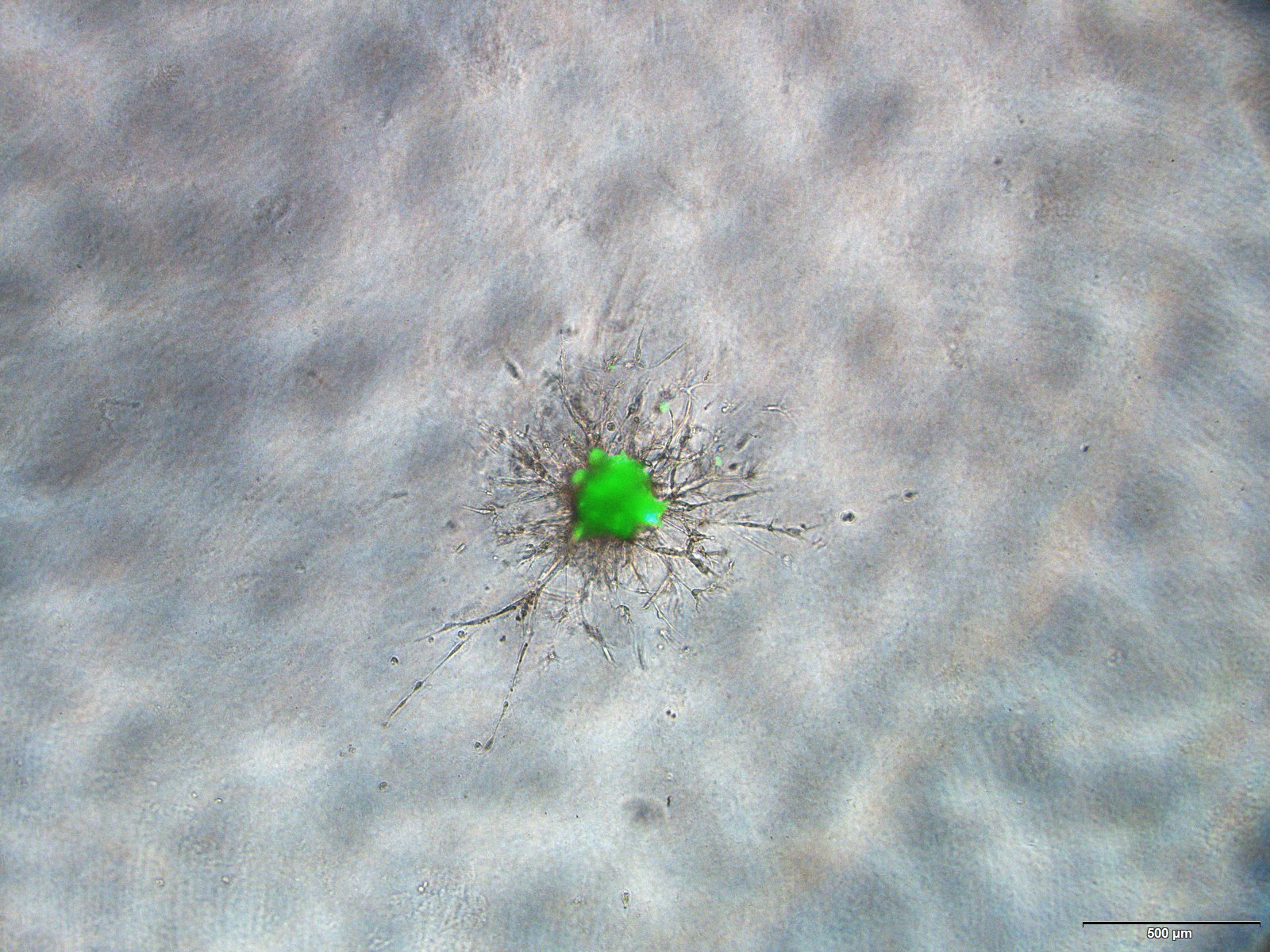

Supplement: Supplementary file 11 — Source Data [file 41467_2020_17139_MOESM11_ESM.zip › Source_Data/Supplementary Figure 4e SNU201 invasion images/SNU201_Vector_2.jpg]

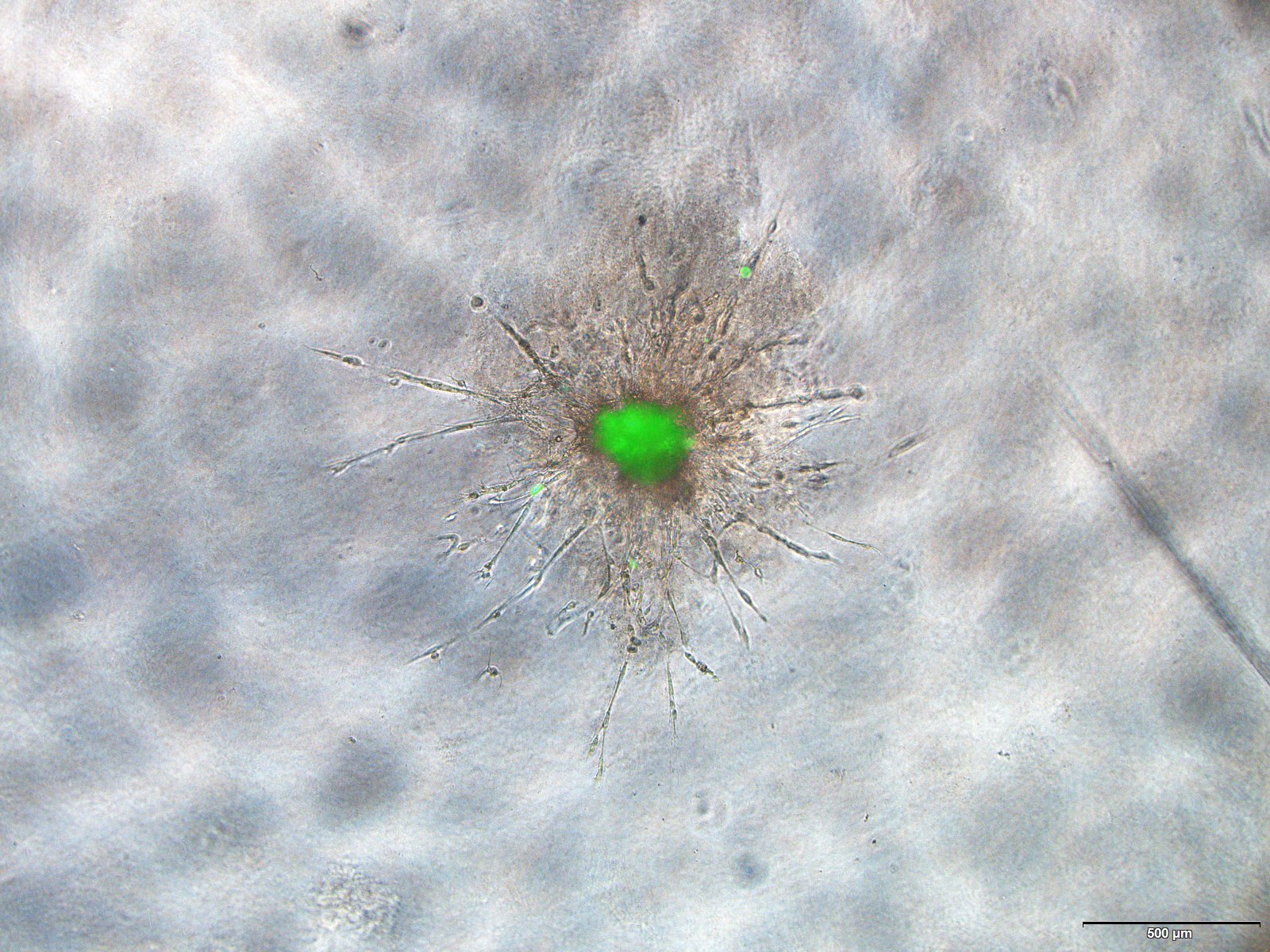

Supplement: Supplementary file 11 — Source Data [file 41467_2020_17139_MOESM11_ESM.zip › Source_Data/Supplementary Figure 4e SNU201 invasion images/SNU201_Vector_3.jpg]
